# Adsorption and Sulfur-Selective Photooxidation of Cysteine on Anatase TiO_2_(101)

**DOI:** 10.1021/jacs.6c07370

**Published:** 2026-06-22

**Authors:** Miguel Blanco Garcia, Daniele Perilli, Chiara Daldossi, Aldo Ugolotti, Daniel Silvan Dolling, Andreas Stierle, Annabella Selloni, Cristiana Di Valentin, Heshmat Noei

**Affiliations:** † Centre for X-ray and Nano Science CXNS, Deutsches Elektronen-Synchrotron DESY, 22603 Hamburg, Germany; ‡ University of Hamburg, Notkestraße 9-11, 22607 Hamburg, Germany; § Department of Materials Science, 9305 University of Milano-Bicocca, Via R. Cozzi 55, I-20125, Milano, Italy; ∥ Department of Chemistry, 6740 Princeton University, Princeton, New Jersey 08544, United States; ⊥ BioNanoMedicine Center NANOMIB, University of Milano-Bicocca, I-20125, Milano, Italy

## Abstract

Understanding how multifunctional amino acids interact with photocatalytic oxide surfaces is essential to controlling their interfacial redox chemistry. Here, we combine scanning tunneling microscopy (STM), X-ray photoelectron spectroscopy (XPS), Fourier-transform infrared reflection absorption spectroscopy (FT-IRRAS), and density functional theory (DFT) calculations to elucidate the adsorption geometry and photooxidation mechanism of cysteine on anatase TiO_2_(101). STM reveals a bridging adsorption motif at the surface Ti sites. XPS and FT-IRRAS indicate that adsorption predominantly occurs in configurations where the amino group remains mostly unprotonated, enabling direct coordination of nitrogen to Ti atoms, while sulfur can also independently interact with Ti atoms. DFT calculations confirm two adsorption configurations with comparable stability: a molecular M­(N,S) structure, where cysteine is bound to the surface through nitrogen and sulfur, and a partially deprotonated DP_COOH_(O,N) structure, where it adsorbs through a carboxylic oxygen and nitrogen. Upon UV irradiation in air, cysteine undergoes highly selective photooxidation at the sulfur site, while the carbon and nitrogen moieties remain largely unaffected. XPS measurements reveal stepwise oxidation of sulfur, progressing from thiol to fully oxidized sulfonic acid (−SO_3_H) through distinct intermediate states. DFT calculations rationalize this selectivity by identifying a surface-mediated mechanism in which molecular oxygen activation promotes sequential sulfur oxidation, consistent with the experimentally observed XPS data. These results demonstrate that adsorption geometry and the local coordination environment at oxide interfaces dictate specific photooxidation pathways, providing a molecular-level framework for controlling selective transformations of multifunctional organic molecules on photocatalytic surfaces.

## Graphical abstract

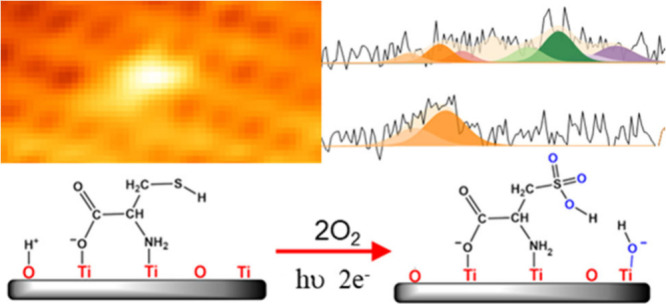

## Introduction

Titanium dioxide (TiO_2_) is one of the most widely studied photocatalytic materials due to its chemical stability, low cost, nontoxicity, and ability to generate reactive oxygen species (ROS) under UV illumination. These ROS, including hydroxyl radicals (·OH) and superoxide (O_2_^–^), enable the oxidative degradation of organic pollutants such as alcohols, dyes, and volatile organic compounds, as well as the inactivation of microorganisms and viruses.^1–6^ For example, recent studies have shown that TiO_2_ effectively degrades SARS-CoV-2 Spike proteins, leading to viral inactivation.^7–9^

Photocatalytic oxidation of simple molecules, such as methanol, isopropanol, and formic acid, has been extensively explored on anatase (101) and rutile (110) surfaces, providing insight into surface reaction pathways.^10–12^ Building on those studies, understanding how biomolecules interact with TiO_2_ is important for improving the performance of sustainable photocatalytic surfaces for air purification, environmental remediation, and antiviral applications.

Among biologically relevant molecules, amino acids are particularly relevant model systems, because they represent the fundamental structural units of proteins. Their adsorption behavior reflects key surface interactions, proton transfer, hydrogen bonding, and coordination to metal sites that ultimately govern the degradation processes of biomolecules. Several studies have examined the adsorption of amino acids, including glycine, alanine, serine, glutamic acid, and others, on TiO_2_ using a combination of X-ray photoelectron spectroscopy (XPS), Fourier-transform infrared spectroscopy (FT-IR), near-edge X-ray absorption fine structure (NEXAFS), and density functional theory (DFT).^13–18^ These studies established common binding configurations such as bidentate (O,O) coordination of the carboxylate group and, in some cases, mixed (O,N) interactions involving the amino group. However, the details depend strongly on the specific TiO_2_ polymorph, surface structure, and the presence of water.

Cysteine represents a particularly important case because its thiol group is highly reactive and plays a key role in redox processes, protein folding, and biochemical signaling.^19–21^ In biological systems, cysteine oxidation follows multiple parallel pathways depending on the local environment and redox conditions,^22^ whereas on solid materials, including metal oxides, oxidation typically proceeds through a ROS-driven stepwise mechanism as described by Reynaud and co-workers.^23^ This sequential SH → SOH → SO_2_H → SO_3_H pathway is central to oxidative protein degradation.^24,25^ Despite this importance, only a limited number of studies have examined cysteine adsorption on TiO_2_, and most of them have focused exclusively on the rutile (110) surface.

Previous studies of cysteine adsorption on rutile TiO_2_(110) generally proposed that the molecule binds through its deprotonated carboxylate group in a bridging (O,O) configuration, a geometry that is stabilized by the close match between the carboxylate O···O distance and the titanium 5-fold coordinated Ti_5c_–Ti_5c_ spacing on the rutile TiO_2_(110) surface.^26–29^ However, the literature contains notable inconsistencies. Ataman et al.^26^ suggested additional S–Ti interactions and even partial C–S bond cleavage, whereas Muir and Idriss^28^ reported only (O,O) and (O,N) geometries without sulfur involvement. Reactive force-field simulations further predicted that the thiol group may participate in hydrogen bonding or proton-transfer processes, adding uncertainty to the adsorption mechanism.^30,31^ A combined STM, XPS, FT-IRRAS, and DFT study showed that cysteine adsorption on rutile is more complex than previously assumed.^32^ At room temperature, three adsorption geometries coexist on the surface: a bidentate carboxylate (O,O) configuration, a mixed (O,N) mode involving amino group coordination, and an (O,S) configuration in which the thiolate interacts with Ti sites. Cysteine can adsorb in either deprotonated or zwitterionic form, and STM revealed dimer formation even at low coverage.^32^

However, the phase of the TiO_2_ surface strongly influences both adsorption and photocatalytic behavior. Rutile (110) exposes rows of fivefold coordinated Ti (Ti_5c_) and twofold oxygen (O_2c_) sites, and surface oxygen vacancies are frequent on this surface, whereas anatase TiO_2_(101) exhibits a zigzag arrangement of Ti_5c_–O_2c_ pairs, a higher tendency for subsurface oxygen vacancy formation, and enhanced charge separation efficiency.^33–36^ These structural and electronic differences lead to distinct adsorption geometries, reaction intermediates, and photochemical reactivity. For example, acetic acid has been observed to adopt mixed adsorption geometries on anatase TiO_2_(101).^37^ Petrik et al. reported that formic acid undergoes temperature-dependent conversion pathways on anatase TiO_2_(101) that have no direct analogue on rutile TiO_2_(110), underscoring the higher configurational flexibility and reactivity of the anatase surface.^38^

Theoretical studies show that anatase TiO_2_(101) stabilizes (O,N) adsorption geometries more effectively than rutile (110), a trend attributed to its larger Ti_5c_–Ti_5c_ spacing (3.79 Å compared with 2.96 Å on rutile).^39,40^ For cysteine specifically, DFT predicts that both (O,O) and (O,N) configurations can be accessible on anatase, with additional stabilization arising from hydrogen bonding involving the thiol group. However, no experimental work has yet verified these adsorption modes on anatase TiO_2_(101) or their photoinduced evolution under UV illumination.

The present work addresses these gaps by providing the first experimental investigation of cysteine adsorption and photocatalytic oxidation on anatase TiO_2_(101). Using STM, XPS, FT-IRRAS, and DFT calculations, we identified the preferred adsorption geometries and protonation states. We further examine how UV irradiation in air drives cysteine oxidation, determine which functional groups undergo chemical transformation, identify the reaction intermediates, and compare these results with our previous studies on rutile (110) surface to reveal the phase-dependent reactivity of TiO_2_. Understanding these processes is essential for advancing the design of photocatalytic materials for self-cleaning and antiviral applications.

## Results and Discussion

In this section, we first present a systematic investigation of the adsorption configurations of cysteine on the anatase TiO_2_(101) surface. This is followed by the analysis of the photooxidation mechanism.

### Nomenclature for Cysteine Adsorption on Anatase TiO_2_(101)

As cysteine contains multiple functional groups and can exist in different protonation states, we first introduce a nomenclature to describe the potential adsorption configurations on the anatase surface. Specifically, we consider the following cases: molecular (M), in which the carboxylic, amino, and thiol groups are in their intact (protonated) forms; deprotonated at the carboxylic or thiol group (DP_COOH_ and DP_SH_, respectively), where the loss of a proton leads to the formation of −COO^–^ or −S^–^; zwitterionic (ZW), in which the proton released from the carboxylic group protonates the amino group, yielding −NH_3_^+^; and bideprotonated (biDP), where both the carboxylic and thiol groups are deprotonated.

Each cysteine adsorption geometry is labeled by the protonation state of the molecule, defined above, followed by a descriptor (in parentheses) indicating which cysteine atoms form covalent bonds with the TiO_2_(101) surface. In all configurations considered, cysteine establishes at least two distinct interactions with surface atoms. These configurations are summarized and schematically illustrated in Scheme 1.

**Scheme 1:**
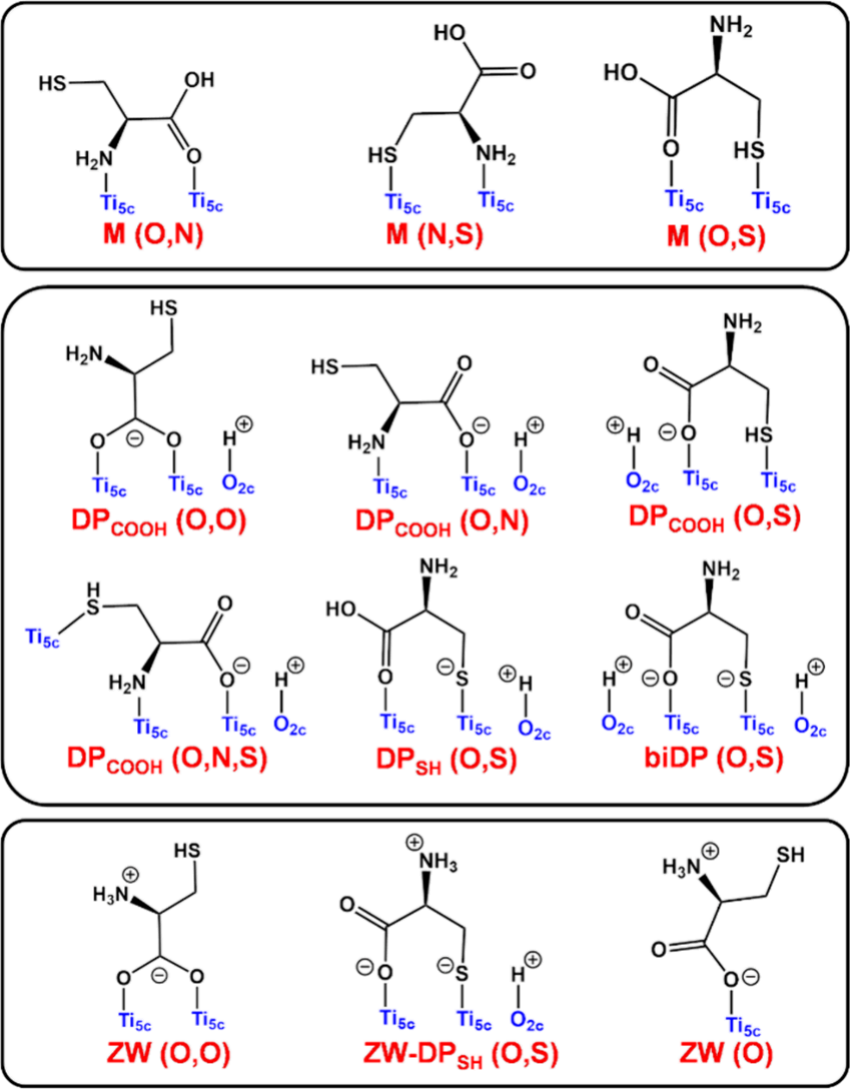
Cysteine Adsorption Modes on Anatase TiO_2_(101)^a^

a The Ti_5c_ and O_2c_ sites indicate undercoordinated surface Ti and O atoms, respectively, which act as anchoring sites for the molecule.

### Adsorption of Cysteine on Anatase TiO_2_(101): Experimental Results

The anatase TiO_2_(101) surface was prepared and characterized under ultrahigh-vacuum (UHV) conditions prior to molecular adsorption. After standard cleaning procedures of sputtering and annealing, the surface structure was examined by scanning tunneling microscopy (STM) to establish a well-defined reference state. Experimental details of the surface preparation are provided in the Supporting Information (SI).

Figure 1a presents a high-resolution STM image of the anatase TiO_2_(101) surface after the deposition of 1 L of cysteine. The surface exhibits a terraced morphology with monatomic step edges. The bright protrusions correspond to alternating Ti_5c_–O_2c_ pairs, which appear as periodic bright features due to electronic contrast differences. Unlike rutile TiO_2_(110), where Ti_5c_ sites are imaged as bright protrusions in empty states, in anatase TiO_2_(101), both Ti_5c_ and O_2c_ contribute to the observed contrast.^33,41^ The crystallographic directions were assigned based on step orientations and island shapes following the approach proposed by Gong et al.,^34^ which relates trapezoidal island morphologies to the surface symmetry. STM images of the clean anatase TiO_2_(101) surface are provided in Figure S2.

**Figure 1:**
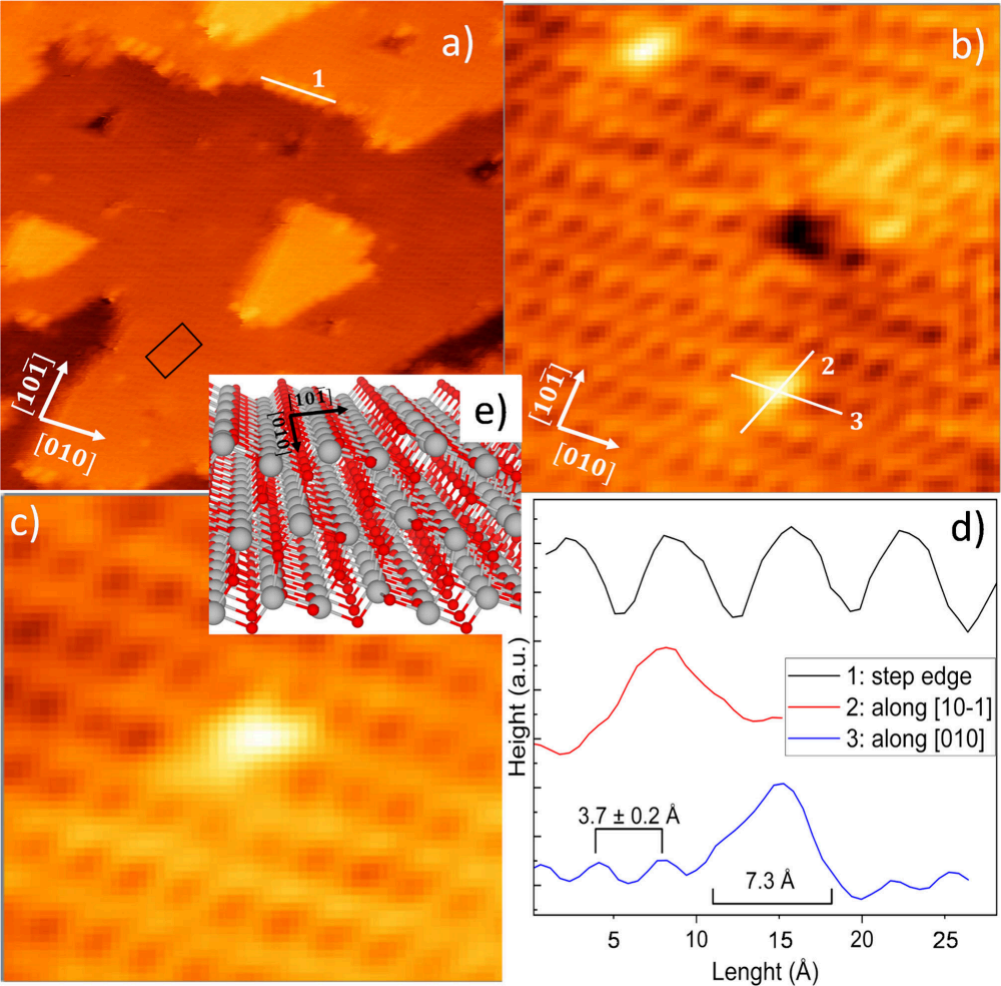
STM images of the anatase TiO_2_(101) surface after dosing 1 L of cysteine at room temperature. All images were acquired in empty state mode with bias voltages between 1 and 1.2 V and tunneling currents of 0.1–0.3 nA. (a) STM image after deposition of 1 L of cysteine (28 × 28 nm^2^). Line scan 1 was taken across the step edge along the [010] direction. (b) Magnified STM image (5 × 5 nm^2^) of Figure 1a, showing two individual cysteine molecules adsorbed on the terrace. Line scans 2 and 3 were taken across these molecules. (c) Close-up STM image (3 × 3 nm^2^) of a single cysteine molecule adsorbed on the anatase TiO_2_(101) terrace, revealing its adsorption atop two Ti_5c_-O_2c_ pairs along [010] and extending toward the next row. (d) Line scans 1, 2, and 3 extracted from Figure 1a and 1b, displaying molecular dimensions and Ti_5c_ spacing. (e) Ball and stick model of the anatase (101) surface Ti in gray, O in red.

Individual cysteine molecules were clearly resolved on the surface. Adsorption is observed both on terraces and preferentially along step edges, suggesting that these sites provide more favorable binding configurations compared with terraces. Preferential adsorption at step edges has been reported before by Setvin et al.,^42^ who demonstrated that these sites act as charge accumulation centers, enhancing their reactivity toward adsorbed molecules. Step edge adsorption has also been reported for other adsorbates such as water^43^ and acetic acid.^44^

Figure 1b, a magnified region of Figure 1a, reveals two individual cysteine molecules adsorbed on a terrace. These molecules appear as bright features, centered on top of two Ti_5c_–O_2c_ pairs and extending toward the adjacent row. This suggests that cysteine is directly bound to two Ti_5c_ sites consistent with a bidentate bridging configuration. The portion extending toward the next row may indicate additional stabilization via hydrogen bonding with surface oxygen atoms, as illustrated in Figure 1c. Line scans 2 and 3, performed on one of these molecules, measure approximately 7 Å in both directions, confirming the expected molecular size of cysteine.^45,46^ Line scan 3 also shows the distance between Ti_5c_–O_2c_ pairs, measured as 7.3 Å, showing that the molecule spans approximately twice this distance, confirming its adsorption across two adjacent Ti_5c_ sites.

STM imaging alone cannot definitively distinguish between different bridging adsorption configurations, such as DP_COOH_(O,N) or M­(N,S), nor can it exclude the presence of other adsorption configurations, since all bridging configurations are expected to produce similar contrast in STM images. For this reason, the STM results are complemented by XPS and FT-IRRAS to further constrain the adsorption mode and the molecule–surface interactions.

XPS measurements were performed on the anatase TiO_2_(101) surface, following cysteine adsorption. Prior to cysteine evaporation, the clean sample showed no carbon, hydroxyl, or water contamination. The O 1s and C 1s core level spectra and the low energy electron diffraction pattern of the clean surface can be seen in Figure S1. Fitting parameters for every XP spectrum are shown in Table S3. After dosing 50 L of cysteine, the surface is nearly saturated with cysteine molecules without any bi- or multilayer formation (Figure S2).

The deconvoluted O 1s core level spectrum (Figure 2a) includes two components belonging to the TiO_2_ structure that are also present in the clean spectra. The peak at 530.3 eV corresponds to lattice oxygen in TiO_2_, while the second component at 531.0 eV has been attributed to either an intrinsic species within the TiO_2_ lattice or asymmetry in the O 1s peak.^47^ For comparison, above the anatase TiO_2_(101) spectrum we plotted the rutile TiO_2_(110) cysteine adsorbed spectrum of our previous study.^32^

**Figure 2:**
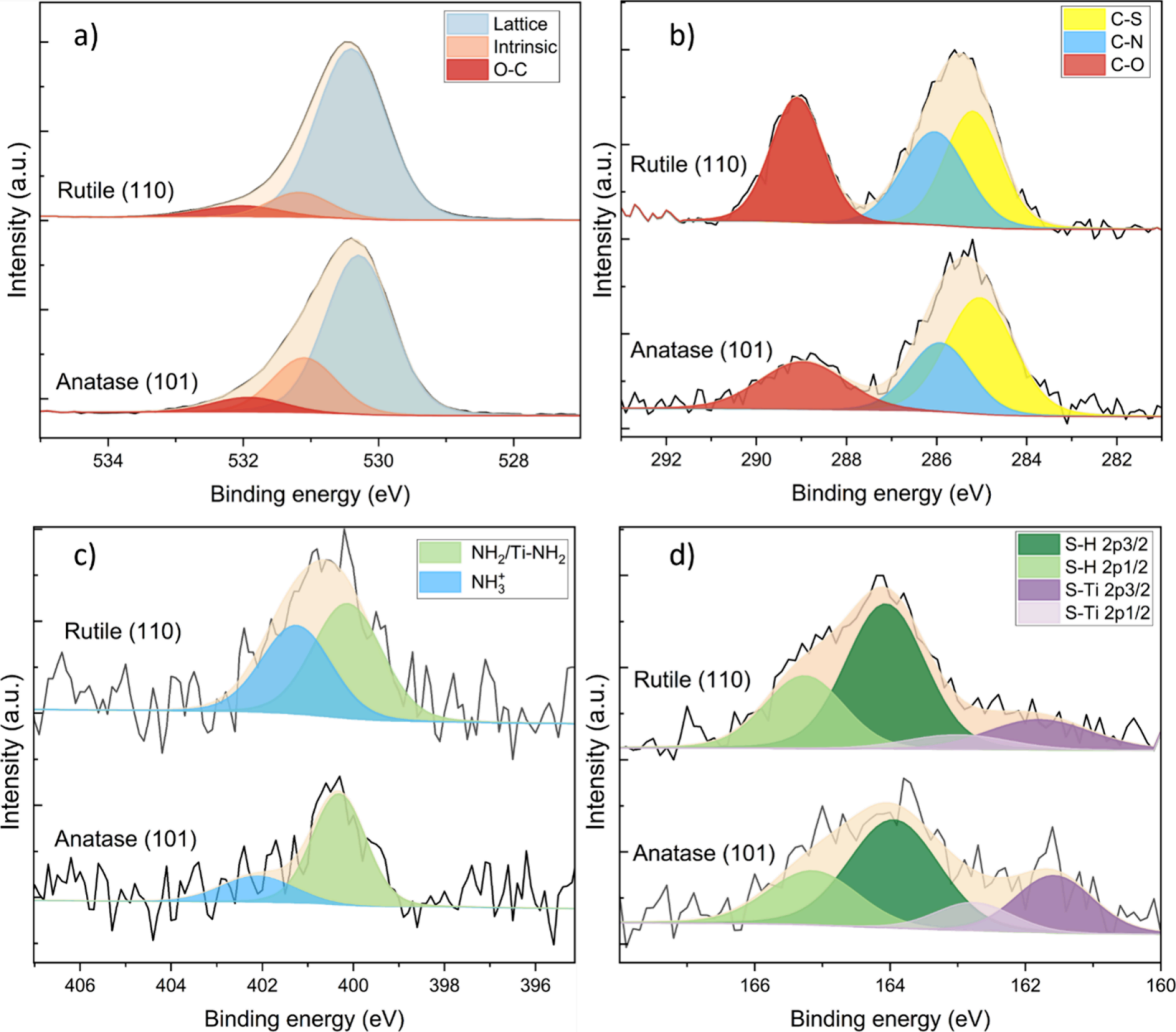
Deconvoluted XP spectra of 50 L of dosed cysteine adsorbed on anatase TiO_2_(101). Experimental data (black lines) and deconvoluted components (colored areas) are presented for (a) O 1s, (b) C 1s, (c) N 1s, and (d) S 2p core levels. For comparison, the corresponding spectra for cysteine adsorbed on rutile TiO_2_(110) from a previous study are shown.

After cysteine evaporation, a new oxygen peak appeared at 532.1 eV, characteristic of oxygen from carboxylic compounds.^26,29,48–51^ Cysteine has two distinct oxygen atoms (C=O, C–OH), which in the case of a protonated carboxylic acid would typically give rise to two O 1s peaks separated by about 1–1.3 eV, with the proton-bonded oxygen appearing at higher binding energy, often above 533 eV.^49,52,53^ In the present spectra, only a single additional O 1s contribution was resolved. While the presence of a single additional O 1s component is commonly associated with carboxylate formation, closely spaced oxygen contributions may be difficult to resolve experimentally because of intrinsic line broadening and the dominance of the intense lattice O 1s peak. In addition, configurations involving hydrogen bonding can shift the binding energies of carboxyl oxygen to lower values, thereby reducing the separation between the two oxygen components. As a consequence, the O 1s spectrum alone does not allow for a definitive distinction between different adsorption geometries.

The deconvoluted C 1s core level spectrum, presented in Figure 2b, reveals three distinct carbon species, corresponding to the three inequivalent carbon atoms in the cysteine molecule. The peak at 289.0 eV, assigned to the carboxyl carbon, appears at the highest binding energy. The peak at 285.9 eV corresponds to the α-carbon (Cα), while the lowest binding energy peak at 285.2 eV is attributed to the carbon bonded to sulfur (C–S).^26,27,54,50^ When comparing these results to those obtained for cysteine adsorption on rutile, no significant differences are observed in the Cα and C–S peak positions or relative intensities. In contrast, a clear difference is observed in the intensity of the carboxyl peak. On rutile, the peak ratio among the three carbon species was approximately 1:1:1, whereas on anatase, the carboxyl peak is significantly weaker than the other two. In principle, contamination or beam induced decomposition could alter peak ratios, but both surfaces were confirmed to be clean prior to dosing and no time dependent changes were observed in the C 1s or O 1s regions after X-ray irradiation, excluding these effects. Similar attenuations of the high binding energy carboxyl component have been reported for other amino acids, such as aspartic acid on Ni(100),^55^ serine on Cu(110),^56^ alanine on Ni(111),^57^ and *p*-aminobenzoic acid on TiO_2_,^58^ and were consistently attributed to photoelectron diffraction, which selectively reduces the detected intensity of specific chemical environments.^59,60^

Figure 2c displays the N 1s core level spectrum, where two distinct components were fitted, exhibiting a peak splitting of approximately 2 eV. This peak position and splitting have been previously reported for various amino acids and structurally related molecules adsorbed on different surfaces.^56,58,61,54,62–65^ The higher binding energy peak at 402.2 eV is attributed to the protonated amino group (−NH_3_^+^), while the lower binding energy peak at 400.3 eV corresponds to the neutral amino group (−NH_2_), which may or may not be coordinated to Ti, as previously demonstrated in rutile by the spectroscopic characterization of CLSs (core level shifts).^32^

A clear difference between anatase and rutile in the N 1s region lies in the relative abundance of the −NH_3_^+^ species. The −NH_3_^+^ peak is much stronger on rutile, although both surfaces were dosed to comparable saturation coverage. This comparison was intentionally performed under saturation conditions, since coverage dependent hydrogen transfer has been shown to influence the distribution of molecular and proton-transferred species on oxide surfaces, as reported for NH_3_ on ZnO.^66^ Therefore, the lower −NH_3_^+^ contribution on anatase is mainly attributed to differences in the adsorption geometry. On anatase, the amino group likely interacts more directly with the TiO_2_ surface through N–Ti coordination, which makes protonation less favorable and reduces the −NH_3_^+^ contribution.

Figure 2d shows the deconvoluted XP spectra of the S 2p core level, which reveal two distinct sulfur species with a peak splitting of 2.3 eV. The lower binding energy peak at 161.6 eV is assigned to sulfur coordinated to Ti_5c_ sites, while the higher binding energy peak at 163.9 eV is associated with the thiol (−SH) group in cysteine. A comparison of the relative peak intensities shows that the S–Ti contribution is more pronounced on anatase than on rutile, indicating that a larger fraction of cysteine molecules adsorb with sulfur directly bonded to the surface. This trend is consistent with the larger Ti–Ti spacing on anatase, which can accommodate sulfur coordination with reduced geometric strain.

We examine these geometries in greater detail in the theoretical section that follows.

Figure 3 shows the evolution of the FT-IRRAS spectra with increasing cysteine coverage on anatase, together with a direct comparison to rutile. If bridging bidentate (O,O) coordination dominates the adsorption of cysteine, two distinct vibrational bands corresponding to the asymmetric (ν_asym_) and symmetric (ν_sym_) stretching modes of the OCO should be observed. Previous studies on carboxylic acids, such as formic acid on anatase TiO_2_(101) and serine on commercial anatase as well as other oxide surfaces indicate that these bands typically appear in the range of 1600–1550 cm^–1^ (ν_asym_) and 1410–1370 cm^–1^ (ν_sym_).^67–69^ On the other hand, adsorption geometries that keep the carbonyl bond (C=O), such as molecular adsorption, are expected to give rise to a distinct C=O stretching vibration in the 1700 cm^–1^ region. We observe a prominent band at 1700 cm^–1^ that is assigned to the carbonyl (C=O) stretching mode, in good agreement with reported literature values for carbonyl vibrations.^70,71^ At 1590 cm^–1^ and 1406 cm^–1^, we identify the ν_asym_(OCO) and ν_sym_(OCO) bands, respectively, aligning well with reported values for deprotonated carboxylic groups.^15,67–69^ Xu et al.^68^ reported a ν_asym_(OCO) mode at 1598 cm^–1^ and a ν_sym_(OCO) mode at 1362 cm^–1^ for formic acid adsorption, while Pászti and Guczi^72^ found a C=O stretching band at 1712 cm^–1^ and asymmetric and symmetric OCO^–^ stretching modes at 1550 cm^–1^ and 1400 cm^–1^, respectively, for glutamic and aspartic acid adsorption on TiO_2_. In comparison, our previous study on rutile (110) revealed slightly shifted bands, with ν_asym_(OCO) at 1585 cm^–1^ and ν_sym_(OCO) at 1363 cm^–1^.^32^

**Figure 3:**
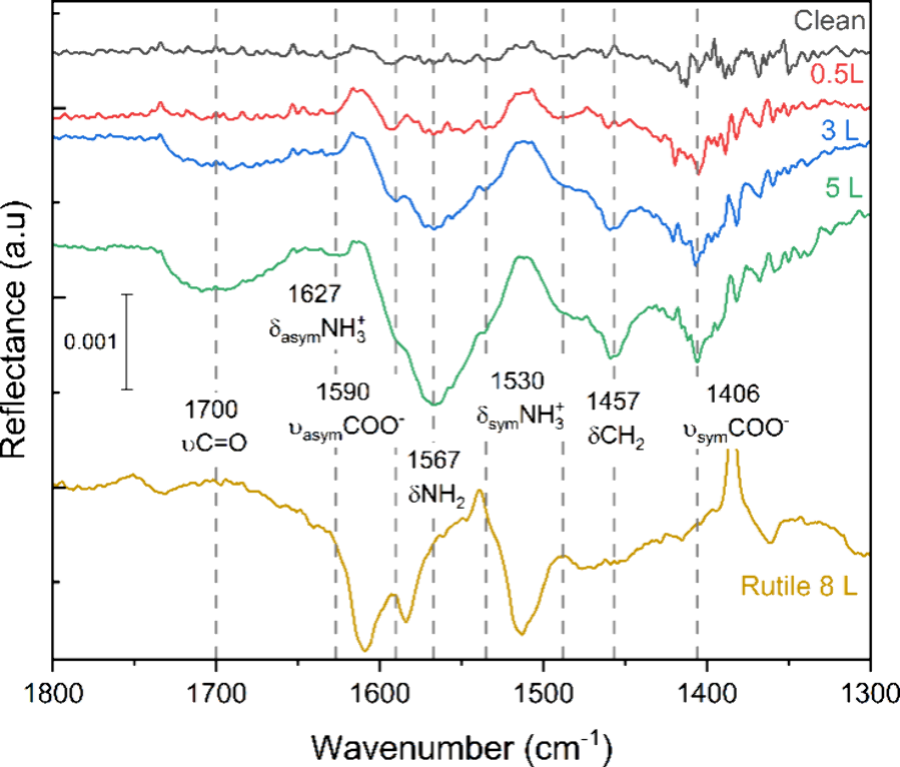
FT-IRRAS spectra of cysteine adsorbed on anatase TiO_2_(101) at room temperature for increasing coverage. The vibrational features of cysteine adsorbed on rutile TiO_2_(110) at 8 L coverage (yellow spectrum) are also included for comparison.

In the N–H region, the −NH_2_ deformation band is clearly observed at 1567 cm^–1^, whereas the −NH_3_^+^ deformation bands at 1627 cm^–1^ and 1530 cm^–1^ are much weaker.^71,73–75^ This indicates that −NH_2_ species predominate with only a minor −NH_3_^+^ contribution, consistent with the trend observed in the N 1s XP spectra. Roddick-Lanzilotta and McQuillan^76^ reported the −NH_3_^+^ asymmetric and symmetric deformation modes at ∼1620 cm^–1^ and ∼1520 cm^–1^, respectively. Ustunol et al.^77^ observed comparable assignments across a range of amino acids on TiO_2_ nanoparticles: C=O stretching at 1740–1710 cm^–1^, ν_asym_(COO^–^) around 1590 cm^–1^, and ν_sym_(COO^–^) around 1410 cm^–1^, the −NH_3_^+^ symmetric and asymmetric deformation bands were observed approximately at 1620 cm^–1^ and 1515 cm^–1^, respectively, while the −NH_2_ deformation appeared at 1560 cm^–1^. In rutile (110), the asymmetric −NH_3_^+^ deformation band is located at 1610 cm^–1^, while the symmetric mode appears at 1513 cm^–1^, and the −NH_2_ deformation at 1550 cm^–1^.^32^ Although the vibrational frequencies are not identical, shifts between anatase and rutile are expected due to differences in their local chemical environments.

Overall, our experimental results lead to three main conclusions. First, STM imaging shows that cysteine adsorbs on top of surface Ti atoms with a bridging bidentate configuration. Second, the N 1s XP spectrum and the N–H vibrational region in IRRAS indicate that the amino group is largely not protonated on anatase; compared with rutile, this hints at adsorption configurations on anatase in which N is directly bonded to Ti. Third, the S 2p XP spectra reveal a larger contribution from sulfur species directly bonded to surface Ti atoms on anatase than on rutile.

### Adsorption of Cysteine on Anatase TiO_2_(101) Surface: Computational Results

To gain deeper insights into the adsorption behavior of cysteine, we used DFT calculations to systematically investigate various adsorption modes and compare their relative energetics to identify the most stable configurations. The most relevant structures were then characterized spectroscopically by calculating core level shift (CLS) values and IR vibrational frequencies, which were directly compared with the experimental data presented above. The slab models employed for the anatase TiO_2_(101) surface are shown in Figure S3.

#### Molecular Cysteine

The most stable molecular adsorption configuration of cysteine on anatase TiO_2_(101), labeled M­(N,S), is shown in Figure 4a. It is characterized by the molecule binding through dative interactions of the −NH_2_ and −SH groups to two Ti_5c_ surface sites, while the −COOH group forms one hydrogen bond with a surface O_2c_ atom. The resulting adsorption energy is −2.08 eV (a negative value indicates that the adsorption is favorable). As reported in Figure S4, several additional molecular adsorption structures were identified, with adsorption energies ranging from −1.43 to −1.89 eV.

**Figure 4:**
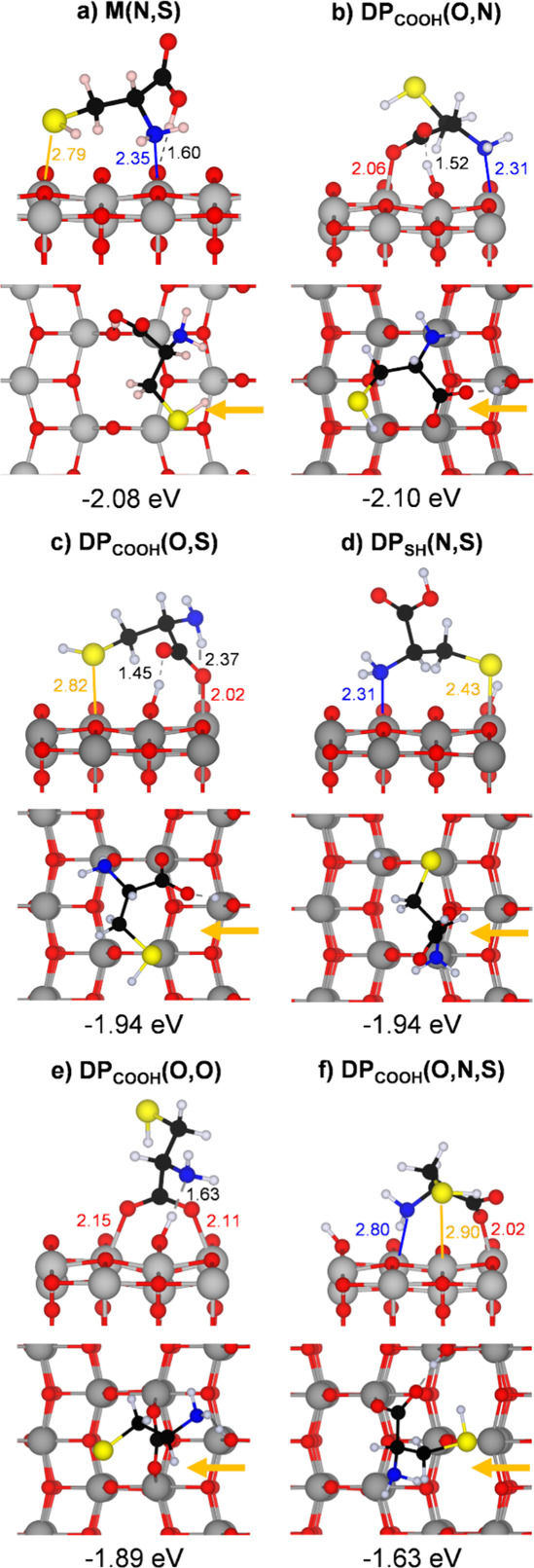
Side and top views of the optimized structures of the most stable molecular and deprotonated cysteine adsorption configurations on the anatase TiO_2_(101) surface, calculated at the PBE+D3+U level. Gray, red, blue, yellow, white, and black spheres represent Ti, O, N, S, H, and C atoms, respectively. Dashed lines indicate hydrogen bonds. Adsorption energies (in eV) are reported below each structure, and selected bond lengths (in Å) are indicated. For clarity, only a portion of the supercell is shown. The orientation of the side view is indicated by an orange arrow in each top view panel.

#### Deprotonated Cysteine

Cysteine contains two acidic groups, −COOH and −SH, that can dissociate on a polar surface such as TiO_2_. Upon adsorption, the carboxylic group typically deprotonates by transferring a proton to a nearby surface bridging oxygen atom, which results in a deprotonated carboxylate species (DP). Adsorption involves coordination of one or both carboxylate oxygen atoms with undercoordinated surface Ti_5c_ atoms. If only one carboxylate oxygen atom binds to the surface, either the amino nitrogen atom or the thiol sulfur atom can coordinate to an adjacent surface Ti_5c_, yielding the DP_COOH_(O,N) and DP_COOH_(O,S) geometries, respectively, as shown in Figure 4b and 4c. Among these three configurations, DP_COOH_(O,N) is the most stable, with an adsorption energy of −2.10 eV, close to that of the M­(N,S) configuration (−2.08 eV), whereas DP_COOH_(O,S) is less favorable (−1.94 eV). Small energy differences between the most stable DP and M structures were also reported by Pantaleone et al. (see Table S1). We note, however, that entropic contributions (not calculated here) tend to favor the deprotonated state over the molecular forms.^39^

In the DP_COOH_(O,N) structure, the interaction between the thiol group and a Ti_5c_ atom on a parallel surface row of Ti_5c_ atoms leads to the tridentate DP_COOH_(O,N,S) structure (Figure 4f). However, due to the strain imposed by the three simultaneous interactions, this configuration is significantly less favorable (−1.63 eV) than the previously discussed adsorption structures, as evidenced by the longer N–Ti and S–Ti bond distances reported in Figure 4f.

When both carboxylate oxygens bind to two Ti_5c_ atoms, the DP_COOH_(O,O) adsorption geometry is formed, as shown in Figure 4e, with a corresponding adsorption energy of −1.89 eV.

Cysteine can also adsorb without involving the carboxylic group in the surface binding, through coordination of both the amino nitrogen and thiol sulfur atoms to surface Ti_5c_ atoms, giving rise to the DP_SH_(N,S) adsorption mode shown in Figure 4d.

All optimized deprotonated adsorption geometries are reported in Figure S5, while the most stable structure within each category, highlighted in bold, is also shown in Figure 4.

#### Zwitterionic Cysteine

Our experimental XPS and FT-IRRAS results indicate that cysteine can adsorb on the surface in either deprotonated or zwitterionic configurations. However, the −NH_3_^+^ signal is significantly weaker than that of −NH_2_, indicating that the zwitterionic form is less frequent. To clarify this point, we also computed the adsorption energy of zwitterionic cysteine on the anatase TiO_2_(101) surface and compared it to those of the molecular and deprotonated forms.

In the zwitterionic configuration, the proton from the carboxylic group is transferred to the amino group rather than to the surface. Protonation of the amino group (−NH_3_^+^) prevents its coordination to a surface Ti_5c_ atom, thereby restricting the adsorption to the (O,O) or (O,S) binding modes. A stable zwitterionic structure ZW-DP_SH_(O,S) is obtained when both the carboxylic and thiol groups become deprotonated with an adsorption energy of −1.81 eV. In this configuration, the proton transferred to the amino group forms one hydrogen bond with a surface O_2c_ site, as shown in Figure 5a, whereas the proton dissociated from the −SH group is transferred to the surface and establishes another hydrogen bond with one of the noncoordinated carboxylate oxygens.

**Figure 5:**
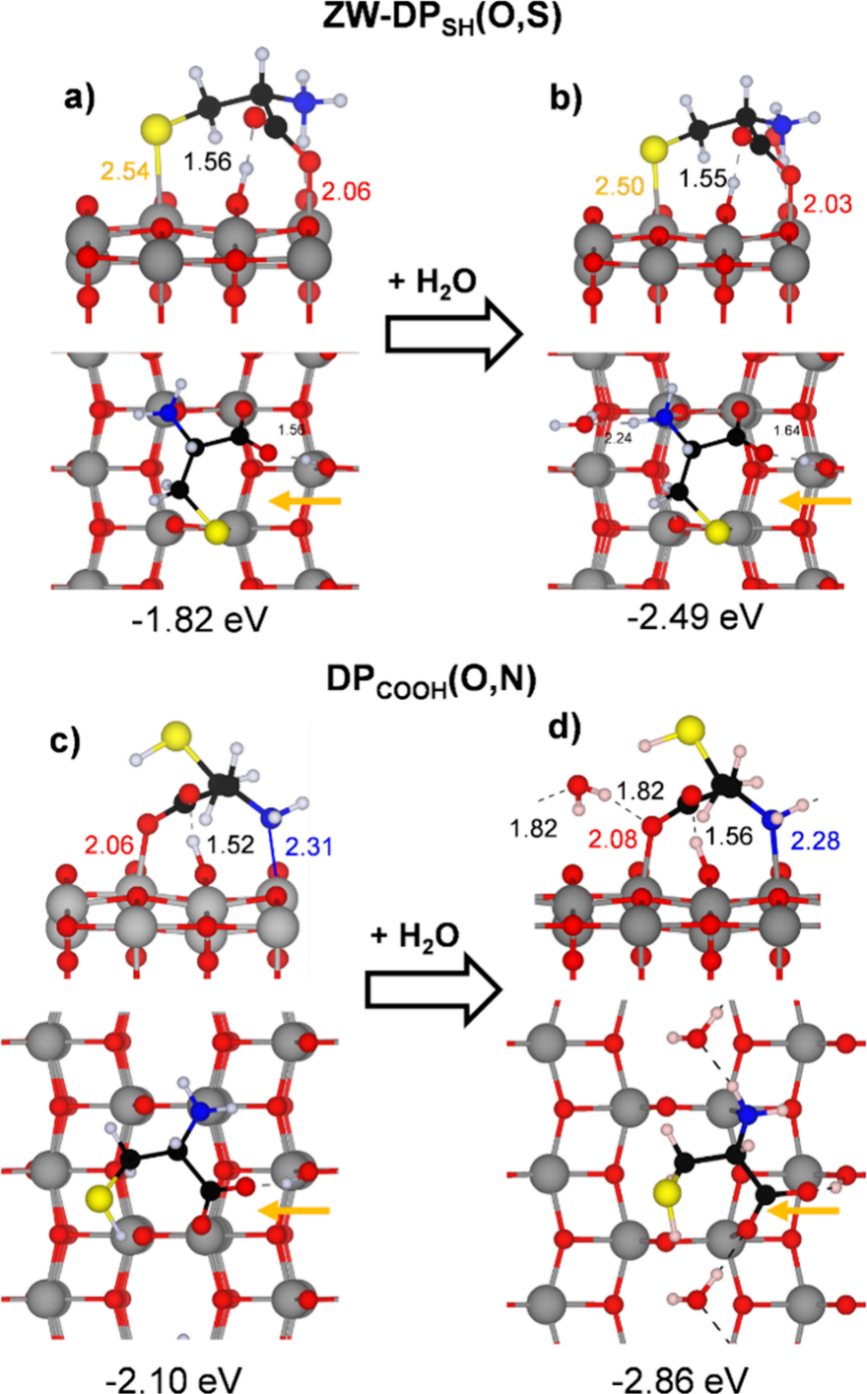
Side and top views of the optimized structures of the most stable zwitterionic (a,b) and deprotonated (c,d) cysteine adsorption configurations on the anatase TiO_2_(101) surface, calculated at the PBE+D3+U level. The right panels show the corresponding optimized structures in the presence of one interacting water molecule. The structure shown in panel c corresponds to that reported in Figure 4b. Gray, red, blue, yellow, white, and black spheres represent Ti, O, N, S, H, and C atoms, respectively. Dashed lines indicate hydrogen bonds. Adsorption energies (in eV) are reported below each structure, and selected bond lengths (in Å) are indicated. For clarity, only a portion of the supercell is shown. The orientation of the side view is indicated by an orange arrow in each top-view panel.

The bidentate ZW­(O,O) structure, reported by Pantaleone et al.^39^ as the most stable configuration based on PBE+D2 calculations, does not correspond to a local minimum within our computational setup (PBE+U+D3; see Table S1 for details). In fact, during structural optimization, the proton spontaneously transfers to an O_2c_ site, yielding a deprotonated form. In our calculations, the preferred zwitterionic structure is monodentate ZW­(O) with the addition of one hydrogen bond between the transferred proton in −NH_3_^+^ and a surface O_2c_ site. We identified two different adsorption ZW­(O) configurations with adsorption energies of −1.57 and −1.73 eV (see Figure S6), which are less favorable than that (−2.10 eV) of the most stable DP_COOH_(O,N). The larger distance between Ti_5c_ atoms on anatase TiO_2_(101) compared to the one on the rutile (110) surface decreases the stability of the bidentate adsorption mode, which could explain why in the zwitterionic form one carboxylate O–Ti_5c_ bond is broken in favor of the formation of a hydrogen bond between the protonated −NH_3_^+^ group and a bridging surface O_2c_ site.

An increased stabilization of the zwitterionic form is expected in the presence of water molecules, which may be available in small amounts even under UHV conditions as in our experiments. To explore this possibility, we added one gas-phase water molecule in our calculations. Among all of our identified hydrated zwitterionic structures (see Figure S6), the most stable one is ZW-DP_SH_(O,S)+1H_2_O, also shown in Figure 5b. However, this configuration is still less energetically favorable than the hydrated deprotonated DP_COOH_(O,N)+1H_2_O structure (Figure 5c,d), as indicated by their adsorption energies of −2.49 and −2.86 eV, respectively. This result, combined with that for anhydrous conditions reported above, and our experimental findings provide a rational basis to explain the limited abundance of zwitterionic species observed experimentally.

#### Spectroscopic Assignment of Adsorption Configurations by DFT Calculations: CLS and IR Vibrational Frequencies

To establish a direct link between the experimentally observed XPS and IR features and specific adsorption geometries, we computed core level shifts (CLSs) and vibrational frequencies for the most relevant configurations. These calculations allow us to assess which adsorption motifs are consistent with the measured spectra and to rationalize differences between molecular, deprotonated, and zwitterionic species. The CLS values for C 1s, N 2s, O 1s, and S 2p are summarized in Table S2.

The experimental O 1s spectrum (Figure 2a) displays a broad feature at ∼532 eV, attributed to oxygen atoms bound to carbon. For gas phase cysteine in molecular form, the two carboxylic oxygens are chemically inequivalent with a calculated CLS difference of 2.1 eV (Table S2), indicating two well separated components.

Upon adsorption, however, the computed splitting was significantly reduced. In the M­(N,S) configuration (Figure 4a), hydrogen bonding between the COOH group and a surface O_2c_ site lowers the CLS difference to 1.5 eV, while in the most stable deprotonated structure, DP_COOH_(O,N) (Figure 4c), the calculated splitting decreases to only 0.2 eV. In both cases, the predicted O 1s components fall within the energy range of the experimentally observed very broad feature (see Figure 2a). These results indicate that the experimentally observed broad O 1s peak is compatible with both geometries while excluding geometries that would generate strongly inequivalent oxygen environments.

In the case of the experimental C 1s spectrum (Figure 2b), three components corresponding to the inequivalent carbon atoms in cysteine are visible, as discussed above. The peak at the highest binding energy, 289.0 eV, is assigned to the carboxyl carbon, followed by the α-carbon (Cα) at 285.9 eV, and the C–S carbon at 285.2 eV. The calculated CLS values (Table S2) reproduce this qualitative trend, with the smallest shift for C–S, an intermediate shift for Cα, and the largest shift for the carboxyl carbon.

The experimental N 1s spectrum (Figure 2c) exhibits two components separated by ∼2 eV. The peak at 402.2 eV corresponds to protonated amino groups (−NH_3_^+^), while the peak at 400.3 eV is assigned to neutral −NH_2_ groups, either coordinated or uncoordinated to Ti_5c_. The calculated CLSs (Table S2), between the nonzwitterionic configurations and the zwitterionic one (ZW-DP_SH_(O,S)), show a similar splitting, and the predominance of the −NH_2_ signal supports the higher stability of the DP_COOH_(O,N) configuration over zwitterionic adsorption.

The experimental S 2p spectrum (Figure 2d) reveals two sulfur species separated by 2.3 eV. The lower binding energy component at 161.6 eV is assigned to sulfur coordinated to Ti_5c_ sites, whereas the higher binding energy peak at 163.9 eV corresponds to protonated thiol (−SH) species. The calculated CLS difference between Ti–S bonded and −SH sulfur atoms is approximately 2 eV (Table S2), in excellent agreement with experiment. Both the molecular M­(N,S) and deprotonated DP_COOH_(O,N) configurations reproduce this splitting. DFT calculations indicate DP_COOH_(O,N) as the energetically preferred configuration, in which the sulfur remains protonated (SH) and does not coordinate to the Ti. This is consistent with the experimental S 2p intensity ratio, which indicates a larger contribution from −SH species compared to Ti–S coordinated sulfur.

To further validate the spectroscopic assignments, we computed the vibrational frequencies for the most stable molecular configuration (M­(N,S), Figure 4a), the three most stable deprotonated configurations (Figure 4b–d), and the most stable zwitterionic configuration (ZW-DP_SH_(O,S), Figure 5a). Comparison with the experimental IR data (Table 1) shows good agreement for both the carboxyl and amino related modes. In particular, the calculated symmetric and asymmetric COO^–^ stretching frequencies of the deprotonated configurations match the experimentally observed bands, whereas the zwitterionic structure predicts distinct features in the NH_3_^+^ region that are not dominant in the spectrum. These results further support the predominance of the deprotonated DP_COOH_(O,N) motif.

**Table 1:** Vibrational Frequencies Assignment (in cm^–1^) Corresponding to Figure 4b–d and Figure 5a^a^

	Theory					Experiments	
Assignment	M(N,S)	DP_COOH_(O,N)	DP_COOH_ (O,O)	DP_COOH_ (O,S)	ZW-DP_SH_(O,S)	Wavenumber	Reference
νC=O	1748	–	–	–	–	1700	^68–70^,^74^,^75^
δ_asym_NH_3_^+^	–	–	–	–	1614	1627	^69^,^71–75^
ν_asym_COO^–^	–	1578	1532	1518	1596	1590	^64^,^65^,^68^,^70^,^75^
δNH_2_	1589	1540	1573	1595	–	1567	^69^,^71–75^
δ_sym_NH_3_^+^	–	–	–	–	1504	1530	^69^,^71–75^
δCH_2_	1423	1407	1411	1425	1423	1457	^74^,^75^
ν_sym_COO^–^	–	1327	1379	1387	1354	1406	^64^,^65^,^68^,^70^,^75^

a Computed values (using PBE+U+D3) are reported for the adsorbed molecular form, the three most stable deprotonated configurations and the zwitterionic one. Modes that are absent in a given structure are indicated with a dash (−).

### Cysteine Photooxidation on the Anatase TiO_2_(101) Surface

#### Cysteine Photooxidation by XPS

To elucidate the progression of cysteine photooxidation, stepwise UV irradiation experiments were performed under atmospheric pressure using exposure times of 5, 15, and 30 min in air, corresponding to early, intermediate, and fully oxidized stages, respectively (further details in the SI). Overall, the XPS data show that photooxidation proceeds selectively at the sulfur moiety, while the carbon and nitrogen regions show no evidence for parallel oxidation. The following section details how this selectivity is reflected across different core levels.

Figure 6a shows the deconvoluted C 1s spectra acquired before and after the UV exposure. Two main spectral changes are observed. First, the component initially assigned to carbon bound to sulfur gradually shifts toward lower binding energies with increasing UV exposure (from 285.2 eV in the pristine state to 284.8 eV after 15 min and 284.6 eV after 30 min). This evolution does not reflect a chemical shift of the C–S bond itself. Instead, as sulfur becomes fully oxidized, the intrinsic C–S contribution is expected to decrease. However, this decrease is masked by the simultaneous accumulation of adventitious carbon during UV irradiation under atmospheric conditions. As exposure time increases, hydrocarbon contaminants adsorb on the surface and contribute strongly to the 284.6–284.8 eV binding energy region, progressively dominating this spectral component and shifting its apparent position toward lower binding energies. As a result, the observed peak no longer represents intact cysteine at extended irradiation times but is largely governed by surface carbon contamination, a well-known effect that is difficult to avoid in ambient-pressure photocatalytic experiments.^78–82^

**Figure 6:**
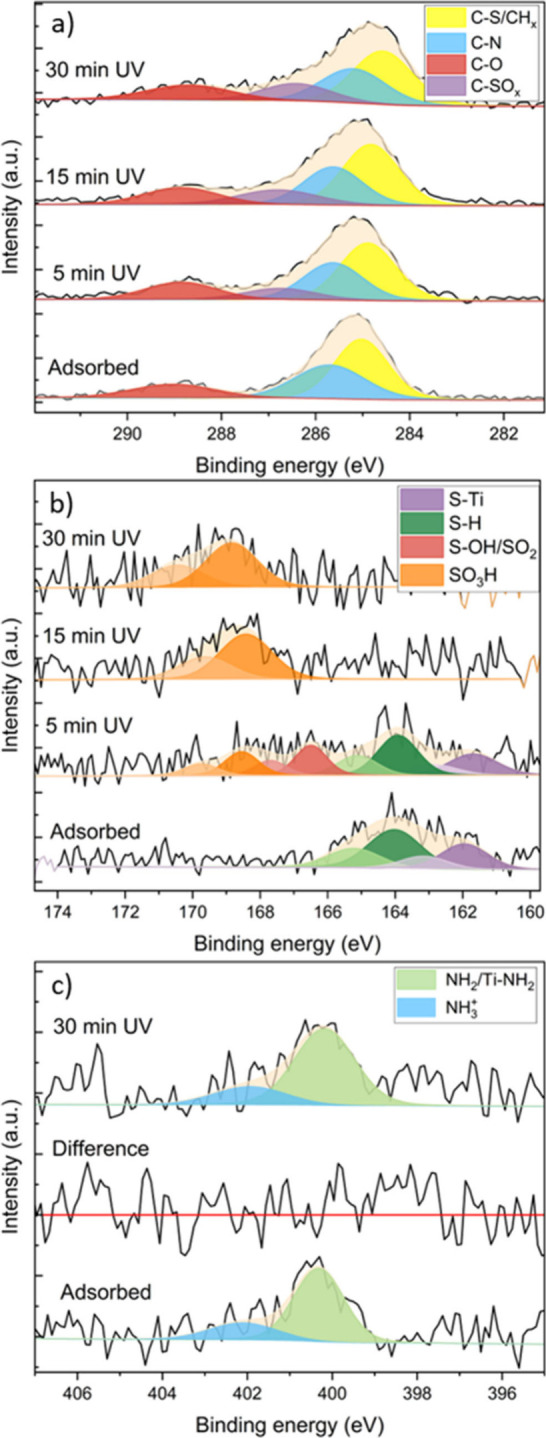
Deconvoluted XP spectra of 50 L of dosed cysteine adsorbed on anatase TiO_2_(101) under UV irradiation. Experimental data (black) and fitted components (colored) are presented for (a) C 1s, (b) S 2p, and (c) N 1s after 5, 15, and 30 min of UV exposure at room temperature and atmospheric pressure. The difference spectrum (30 min – adsorbed) in (c) confirms the absence of changes in nitrogen species.

The second significant feature is the emergence of a new peak at 286.6 eV, which grows with coverage and falls within the binding energy range of carbon bonded to sulfate or sulfite groups. Instead, the alternative possibility of carbon bonded to nitro species can be ruled out, as Figure S8 (N 1s spectrum) shows no evidence of NO_*x*_-related species, which would always appear higher than 403 eV.^83–86^ Additionally, the absence of any noticeable change in the N 1s peak suggests that oxidation does not occur at the nitrogen site.

To further verify that the oxidation is photon driven, we performed a control experiment in the dark. A cysteine-covered surface was exposed to ambient conditions for 30 min without illumination (Figure S7). Under these conditions, no new C–SO_*x*_ component appeared in the C 1s region, and no changes were observed in the S 2p spectra, confirming that cysteine oxidation does not occur without photon excitation. On the other hand, the low binding energy growth of the C–S peak was still observed in the dark, consistent with adventitious carbon accumulation rather than chemical transformation.

A pronounced transformation is observed in the S 2p spectrum (Figure 6b), where the S–H (163.9 eV) and S–Ti (161.6 eV) peaks disappear with increasing UV exposure, and two new sulfur species emerge at approximately 166.5 and 168.7 eV. Oxidation of cysteine at the sulfur site has been reported to involve, depending on the chemical environment, a sequence of sulfur oxyacid species including sulphenic (−SOH) and sulphinic (−SO_2_H) acids before reaching the fully oxidized sulfuric form (−SO_3_H), also known as cysteic acid.^21,22^

After 5 min of exposure, a new peak at 166.5 eV appears, which is consistent with intermediate oxidation states such as sulfenic (−SOH) and sulphinic (−SO_2_H) acids and an emerging peak at 168.7 eV. However, at longer UV exposure times (15 and 30 min), also the 166.5 eV peak disappears, leaving only the 168.7 eV peak, which is consistent with the fully oxidized sulfonic (−SO_3_H) species. The lack of spectral changes between 15 and 30 min suggests that the sample reaches complete oxidation within the first 15 min of UV exposure.

This behavior is in line with the oxidation pathways proposed for the electrochemical oxidation of cystine in water in a previous study.^23^ The latter identified both sulphenic (−SOH) and sulfonic acid (−SO_3_H) species, reporting approximately 3 eV binding energy separation between them. In contrast, our data show a splitting of 2 eV, suggesting that the peak at 166.5 eV is likely a mixture of sulphenic (−SOH) and sulphinic (−SO_2_H) species. Further supporting this peak assignment, studies on sulfonic acid-functionalized carbon nanotubes reported an S 2p peak at 169.5 eV, attributed to R–SO_3_H species, aligning closely with the peak we observed at 168.7 eV. However, while these comparisons support the proposed peak assignments, the microscopic reaction mechanism and the nature of the reactive intermediates involved under photocatalytic conditions remain unclear for the present system.

The O 1s region (Figure S8) was also examined before and after 30 min of UV irradiation. No significant changes were observed, as the signal is dominated by lattice oxygen from TiO_2_, masking contributions from oxidized adsorbates. Therefore, the oxidation process is primarily assessed by using the more chemically sensitive C 1s and S 2p regions.

Although cysteine oxidation can follow different pathways depending on the environment, the UV experiments presented here show selective transformation of the thiol (−SH) group and a progressive shift of the S 2p signal toward higher binding energy, consistent with stepwise increases in the sulfur oxidation state up to −SO_3_H. To rationalize these spectral changes and identify the underlying elementary steps, we combined the experimental XPS observations with DFT calculations of the photooxidation pathway.

#### Cysteine Photooxidation: DFT Calculations

The photooxidation pathway was modeled in the triplet excited state at the HSE06+D3 level, starting from the experimentally observed and energetically favored DP_COOH_(O,N) adsorption configuration. The relative stability ordering of the different adsorption structures predicted by PBE+U+D3 was confirmed at the HSE06+D3 level of theory (see Table S1), further justifying the use of the optimized DP_COOH_(O,N) as the initial structure (Figure 4c). The calculations traced the elementary steps from the adsorbed thiol to the fully oxidized sulfonic acid, enabling direct comparison between computed intermediates and the XPS evolution observed under UV irradiation. Figure 7 summarizes the proposed mechanism. The reaction is initiated by physisorption of O_2_ at a Ti_5c_ site (**II**), followed by a photoactivated step that forms a chemisorbed superoxo species accompanied by −SH deprotonation and sulfur oxidation (**III**). This step has a small electronic energy cost (+0.44 eV), with the photoexcited electron localizing on O_2_ (to form O_2_^–^) and the hole on the sulfur atom. As a result, the oxidation state of sulfur increases from −2 to −1, consistent with the initial upward shift of the S 2p binding energy observed experimentally after 5 min UV exposure.

**Figure 7:**
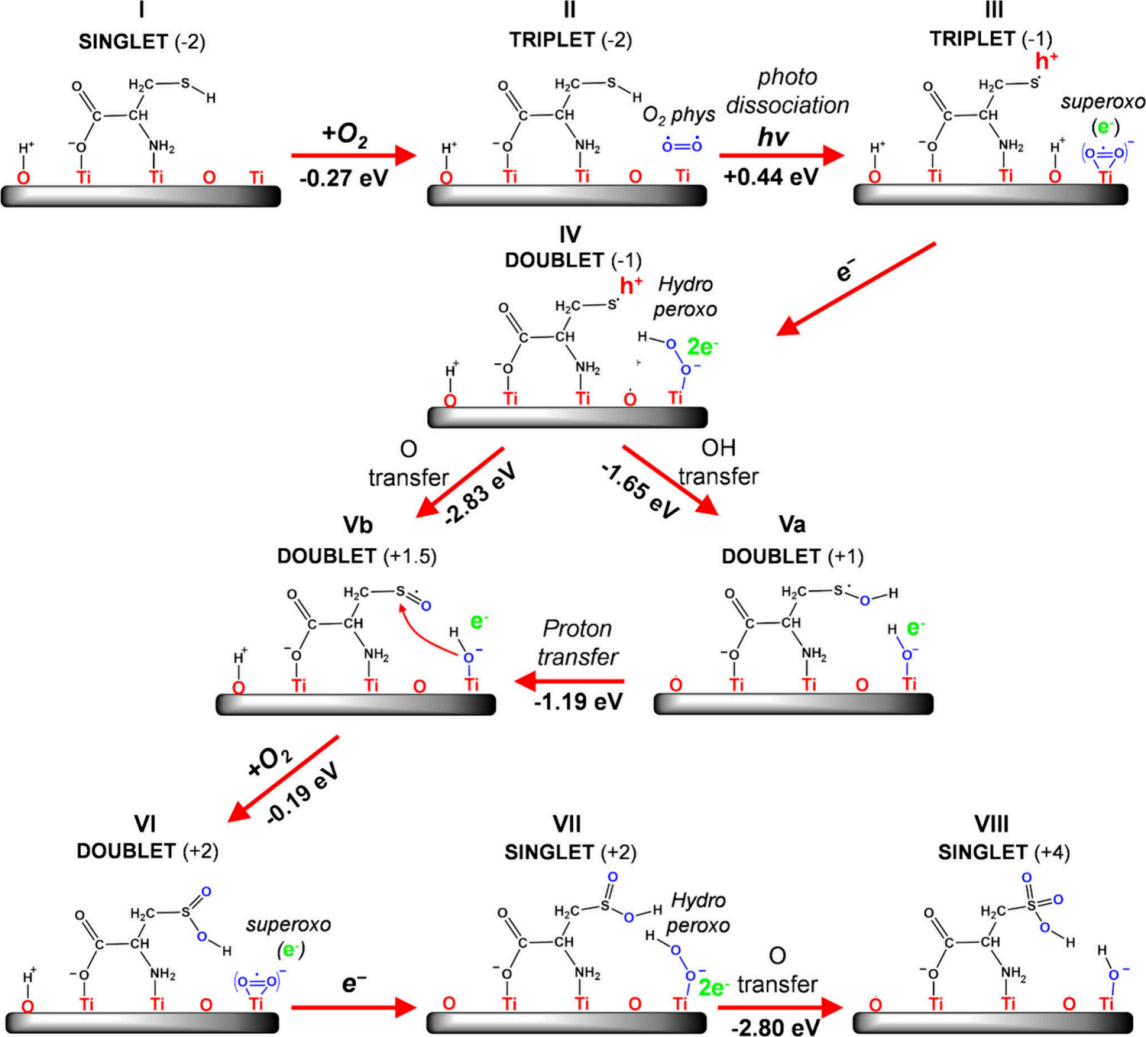
Intermediates of cysteine photooxidation on anatase TiO_2_(101) in the presence of O_2_. For each intermediate (labeled **I, II**, etc.), the spin multiplicity (singlet, doublet, etc.) of the system is reported, with the oxidation state of sulfur in parentheses. Two reaction steps involve excess electrons transferred from the anatase TiO_2_ bulk, which are balanced by surface protons to maintain overall charge neutrality (e^–^ + H^+^) of the periodic model. Oxidation steps are induced by photoexcited holes or hydroperoxide species formed upon transfer of photoexcited electrons and excess bulk electrons to molecular O_2_. The number and localization of photoexcited electrons and conduction electrons from bulk anatase TiO_2_ involved in the photooxidation process are shown in green. Unpaired electrons are indicated by dots. Energies are computed at the hybrid functional HSE06 level with D3 corrections. Further details on the intermediate structures are reported in Figure S9.

Next, we assume that the superoxo intermediate is further reduced by excess electrons from bulk (reduced) anatase TiO_2_, which are readily available due to native oxygen vacancies or other intrinsic defects. This reduction leads to the formation of a peroxo species that is stabilized by protonation to form an adsorbed hydroperoxo (OOH) on the TiO_2_ surface (**IV**). This highly reactive intermediate drives subsequent sulfur oxidation, either via sequential OH and proton transfer (**Va**) or through direct O transfer to form −S=O (**Vb**), which is energetically preferred (−2.83 eV). Because of this oxidation, the sulfur oxidation state increases, to approximately +1 for −S(·)–OH and +1.5 for −S=O(·). In principle, oxidation states of 0 and +1 are expected for −S–OH and −S=O, respectively; however, in both cases sulfur is effectively more oxidized by the photoinduced hole, as evidenced by the presence of an unpaired electron localized on sulfur in −S(·)–OH and partially localized on the sulfur atom of the −S=O(·) moiety.

To complete the oxidation process, a second O_2_ molecule is required. This adsorbs on a Ti_5c_ site while the terminal OH^–^ at that site is transferred to the −S=O group to form a −SOOH intermediate (overall −0.19 eV), further oxidizing sulfur to an oxidation state of +2 (**VI**). Subsequent reduction of the newly adsorbed O_2_ (**VII**) and oxygen transfer ultimately yield the fully oxidized product, −SO_3_H (**VIII**), with a substantial energy gain (−2.80 eV).

The photooxidation mechanism described above requires one photon, two O_2_ molecules, and two TiO_2_ excess electrons to fully oxidize one cysteine −SH group to a sulfonic acid, while forming a chemisorbed OH^–^ species. The overall charge and mass balanced reaction can be summarized as

1 $$\mathrm{COOH-CH-}{\mathrm{NH}}_{2}{\mathrm{-CH}}_{2}\mathrm{-SH}\;\left(\mathrm{cysteine}\right)+2O_{2}+h\upsilon +2e^{-}\left(\mathrm{bulk}\right)\to {\mathrm{COO}}^{-}\mathrm{-CH-}{\mathrm{NH}}_{2}{\mathrm{-CH}}_{2}{\mathrm{-SO}}_{3}H+{\mathrm{OH}}^{-}$$

To connect the computed intermediates with the experimentally observed evolution of the S 2p region under UV irradiation, we evaluated sulfur 2p level shifts along the oxidation pathway, from thiol (formal oxidation state −2) to sulfonic acid (+4). The calculations predict a monotonic increase in S 2p binding energy with increasing sulfur oxidation state, in good agreement with the ∼5 eV shift observed experimentally (Table 2). The only exception is the SOH intermediate (**Va**); since in the computed energy profile (Figure 7) this intermediate is less favorable than the direct formation of the −SO species (**Vb**) via oxygen transfer, we may conclude that the stepwise oxidation pathway likely bypasses the −SOH intermediate. In our calculations, the overall variation of sulfur’s CLSs is 5.5 eV, in good agreement with the experimentally observed increase in the XPS binding energy of ∼5 eV.

**Table 2:** Formal Oxidation States (OS) of S, as Determined from the Structural Formula, Energy Positions of the S 2p States Obtained from the Projected Density of States, and Corresponding Core Level Shifts (CLS), Defined as the Difference in S 2p Energy Relative to the −SH Reference Configuration, for the Computed Cysteine Oxidation Intermediates Reported in Figure 7

Intermediate	S group	OS	S 2p (eV)	CLS (eV)
***I***	–*SH*	–2	–161.2	0.0
***III***	–*S*	–1	–162.3	–1.1
***Va***	–*SOH*	+1	–165.0	–3.8
***Vb***	–*SO*	+1.5	–164.2	–3.0
***VI***	–*SO*_*2*_ *H*	+2	–164.4	–3.2
***VIII***	–*SO*_*3*_ *H*	+4	–166.7	–5.5

With regard to the second most stable cysteine configuration, M­(N,S), it is plausible that this species may undergo photoinduced dissociation of the carboxylic group. Such a process could drive the system toward the formation of the more stable DP_COOH_(O,N) configuration. This intermediate may then undergo further oxidation following the pathway discussed above.

Finally, the experimental N 1s core level spectra indicate that the amino group remains unchanged during UV irradiation. To rationalize this selectivity, we examined a competing pathway in which the −NH_2_ group is oxidized to −N–OH following formation of the superoxo/hydroperoxo species (Figure S10). This process is energetically less favorable (−1.19 eV) than sulfur oxidation via direct oxygen transfer (−2.83 eV), providing a thermodynamic explanation for the experimentally observed preference for thiol oxidation.

## Conclusions

In this work, we combined STM, XPS, FT-IRRAS, and DFT calculations to elucidate the adsorption and photooxidation behavior of cysteine on the anatase TiO_2_(101) surface. STM images reveal that cysteine adsorbs in a bridging configuration on surface Ti sites, occurring both on terraces and preferentially at step edges. Spectroscopic data indicate that, under UHV conditions, the amino group is predominantly unprotonated on anatase, in contrast to rutile TiO_2_(110), and that a significant fraction of molecules interact directly with the surface via the nitrogen and sulfur atoms.

DFT calculations allow us to rationalize these observations by identifying two energetically favored and nearly degenerate adsorption configurations: a molecular M­(N,S) structure, in which cysteine binds through the amino and thiol groups, and a deprotonated DP_COOH_(O,N) configuration, where the carboxylate and amino groups coordinate directly to surface Ti sites. The close energetic competition between these geometries explains the simultaneous presence of molecular and deprotonated species, as inferred from the experimental data.

Upon UV irradiation in air, cysteine undergoes selective photooxidation at the sulfur site. XPS reveals the disappearance of thiol −SH and S–Ti components, along with the appearance of S 2p features at higher binding energies, consistent with stepwise sulfur oxidation. DFT calculations provide a mechanistic picture of this behavior, showing that photoactivated oxygen species on anatase preferentially oxidize the thiol group through a stepwise pathway involving oxygen-transfer reactions, ultimately yielding the fully oxidized sulfonic acid (−SO_3_H). The computed energetics account for both the observed sequence of sulfur oxidation states and the absence of competing oxidation at the amino group.

Overall, this study presents a comprehensive experimental and theoretical investigation of cysteine adsorption and sulfur-selective photooxidation on anatase TiO_2_(101). By combining surface-sensitive spectroscopy, microscopy, and DFT calculations, we identify the most stable adsorption configurations and elucidate the microscopic pathways governing cysteine photooxidation. These findings advance fundamental insight into amino acid reactivity on TiO_2_ surfaces, with relevance for photocatalysis and biomolecule-oxide interface chemistry.

## Supplementary Material



## Notes

Crystal preparation methods, cysteine evaporation details, experimental procedures for X-ray photoelectron spectroscopy (XPS), scanning tunneling microscopy (STM), and Fourier-transform infrared reflection absorption spectroscopy (FT-IRRAS); thickness estimation procedure; detailed computational methodology including density functional theory (DFT) calculations, adsorption energy evaluations, and core level shift calculations; additional figures and tables illustrating surface characterization, calculated binding geometries, adsorption energies, and core level shifts. (PDF)

## Abbreviations

TiO_2_: Titanium dioxide
cys: cysteine
XPS: X-ray photoelectron spectroscopy
FT-IRRAS: Fourier-transform infrared reflection absorption spectroscopy
STM: scanning tunneling microscopy
DFT: density functional theory
SARS-CoV-2: severe acute respiratory syndrome coronavirus 2
CLS: core level shift
LEED: low energy electron diffraction
DP: deprotonated
ZW: zwitterionic
