# Supplementary material for: Adsorption and Sulfur-Selective Photooxidation of Cysteine on Anatase TiO2(101)
Source: J Am Chem Soc. 2026 Jun 22;148(25):26710–23. doi: 10.1021/jacs.6c07370 (PMC13339627; doi:10.1021/jacs.6c07370)
Supplement: Supplementary file 1 [file ja6c07370_si_001.pdf]

Supporting Materials for

Adsorption and Sulphur-Selective Photooxidation of Cysteine on Anatase TiO_2(101)

Miguel Blanco Garcia[1,2], Daniele Perilli[3], Chiara Daldossi[3], Aldo Ugolotti[3], Daniel Silvan Dolling[1,2], Andreas Stierle[1,2], Annabella Selloni[4], Cristiana Di Valentin[3,5]*, Heshmat Noei[1]*

AUTHOR ADDRESS

[1] Centre for X-ray and Nano Science CXNS, Deutsches Elektronen-Synchrotron DESY, 22603 Hamburg, Germany

[2] University of Hamburg, Notkestraße 9-11, 22607 Hamburg, Germany

[3] Department of Materials Science, University of Milano-Bicocca, Via R. Cozzi 55, I-20125, Milano, Italy

[4] Department of Chemistry, Princeton University, Princeton, New Jersey 08544, United States

[5] BioNanoMedicine Center NANOMIB, University of Milano-Bicocca, I-20125, Milano, Italy

* Email: cristiana.divalentin@unimib.it and heshmat.noei@desy.de

S.I. 1 EXPERIMENTAL AND COMPUTATIONAL DETAILS

1.1 Crystal Preparation methods:

The single crystalline anatase TiO_2 (101) sample, 8 mm × 8 mm × 2 mm (Surface Net Ltd.), was subjected to multiple cycles of argon ion sputtering (1 keV, 10^{-6} mbar Ar^+, 5 minutes) and subsequent annealing. The annealing process was carried out at 700 K under back pressure of 10^{-6} mbar of O_2 for 10 minutes, followed by an additional 10 minutes annealing under ultra-high vacuum (UHV) condition at 950 K. This careful preparation resulted in a flat and uncontaminated anatase surface as reported by (*1*) The surface was prepared until a clean, well-defined (1 x 1) low energy electron diffraction (LEED) pattern was achieved, and XPS analysis confirmed the absence of contaminants in O 1s and C 1s core levels.

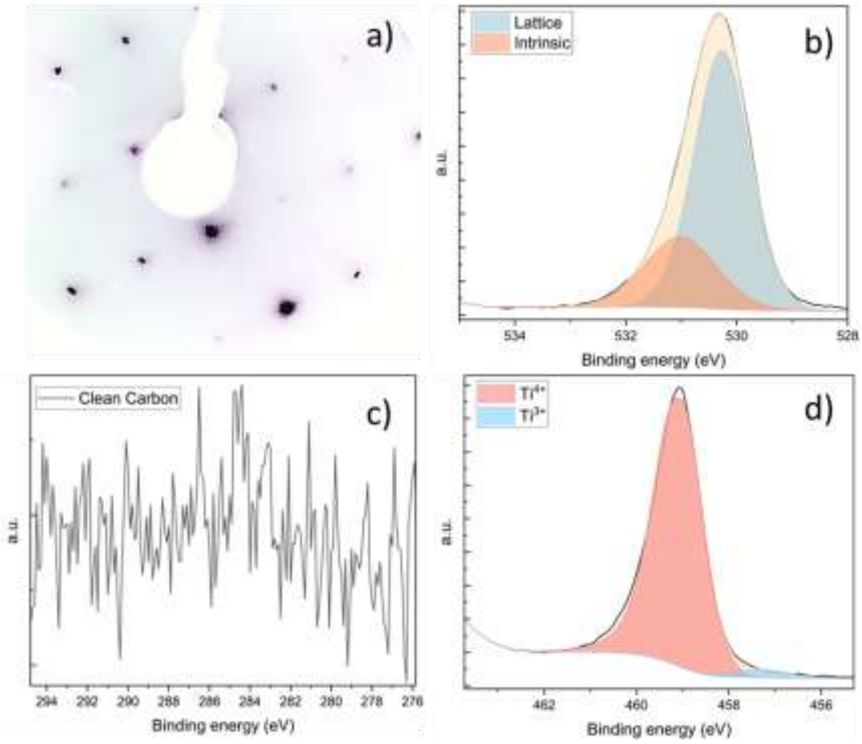

Figure S1. (a) LEED pattern of anatase TiO_2(101) after preparation under UHV taken at 100 eV. Experimental and deconvoluted XP spectra of the clean anatase (101) surface (b) O 1s, (c) C 1s, (d) Ti 2p 3/2.

1.2 Evaporation of cysteine:

Cysteine evaporation was performed in two different UHV sample preparation chambers. For XPS and STM measurements, evaporation occurred in a load lock connected to the STM chamber, with a base pressure in the low 10^{-9} mbar range. During XPS measurements, the sample had to be transferred post-evaporation through a tunnel connecting all equipment, maintaining a base pressure of low 10^{-9} mbar, requiring around 15 minutes for the full transfer process.

The FT-IRRAS chamber, also with a base pressure of low 10^{-9} mbar, is equipped with an evaporation line and a nozzle directed at the sample, allowing deposition and measurement without relocating the sample from the measurement stage.

For all experiments, the gas line containing cysteine was pumped, baked out, and heated to 130 °C during exposure. Temperature measurements were taken using a type K thermocouple attached to the glass tube.

1.3 X-ray photoelectron spectroscopy:

All XPS measurements were conducted at the DESY Nanolab at the Centre for X-ray and Nano Science, DESY, Hamburg (*2*). The instrument is equipped with a monochromated Al K_α source (1.486 keV) and a Phoibos 150 hemispherical energy analyzer, operating under a base pressure of low 10^{-10} mbar. Data analysis was performed using CasaXPS software.

1.4 Scanning tunneling microscopy:

STM images were acquired using a combined STM/AFM system under UHV conditions at a pressure of low 10^{-11} mbar. Measurements were performed in constant current mode at room temperature, employing a tungsten tip. Scanning parameters included voltage ranges from 0.8 V to 1.2 V and current ranges from 0.1 nA to 0.3 nA.

1.5 Fourier-Transformed Infrared Spectroscopy:

FT-IRRAS spectrometer (Bruker Vertex V80) is connected to a UHV chamber with a base pressure of low 10^{-9} mbar. Each IR spectrum was taken in reflection mode with 512 scans at a resolution of 2 cm^{-1} using nonpolarized light.

1.6 Thickness estimation:

The thickness of the cysteine layer (d_{meas}) was estimated by calculating the area intensity of the TiO_2 $2p_{3/2}$ peak using Formula 1.

$$I_M = I_0 \cdot e^{\frac{-d_{meas}}{\lambda}}$$

In this formula, I_M represents the area of the Ti_{2p} 3/2 peak in anatase $TiO_2(101)$ after cysteine adsorption, I_0 denotes the area of the same peak for the clean surface, and λ stands for the effective attenuation length (EAL) of electrons in L-cysteine, extracted from the NIST Database 82 (*3*)). The electrons in Ti $2p_{2/3}$ have a kinetic energy of 1027.56 eV and have an asymmetry parameter (β) of 1.41. We consider the stoichiometric coefficients for cysteine: H→7, C→3, N→1, O→2, S→1; it has 42 valence electrons, a band-gap energy of 4.62 eV and a density of 1.68 g/cm^3. (*4*). The incidence and emission angles of the XPS set up are 55^0 and 0^0, correspondingly.

The average EAL for Ti $2p_{3/2}$ Ek = 1027.56 eV is recorded as EAL = 27.4 Å, providing a practical average EAL value applicable for thicknesses up to 5 nm. I_0 has a value of 44300 counts per seconds cps and I_m has 38000 cps.

The calculated total thickness approximates to 0.4 nm, signifying nearly half a monolayer of cysteine, assuming its dimensions as 6.4 Å in length and 3.7 Å in width (*5*). This implies that even after dosing 500 L of molecules, a saturation level is attained that impedes the formation of multilayers at standard room temperature.

Figure S2 presents a STM image of an anatase (101) surface nearly fully saturated with 50 L of cysteine. At this coverage the whole surface is fully covered, however we do not observe any bi- or multilayer formations as it was also calculated in XPS. This suggests that there is a limit below a monolayer in which cysteine can adsorb on TiO_2 at room temperature. At this coverage, no distinct preferred orientation or ordered molecular layer of cysteine can be discerned on the surface.

(

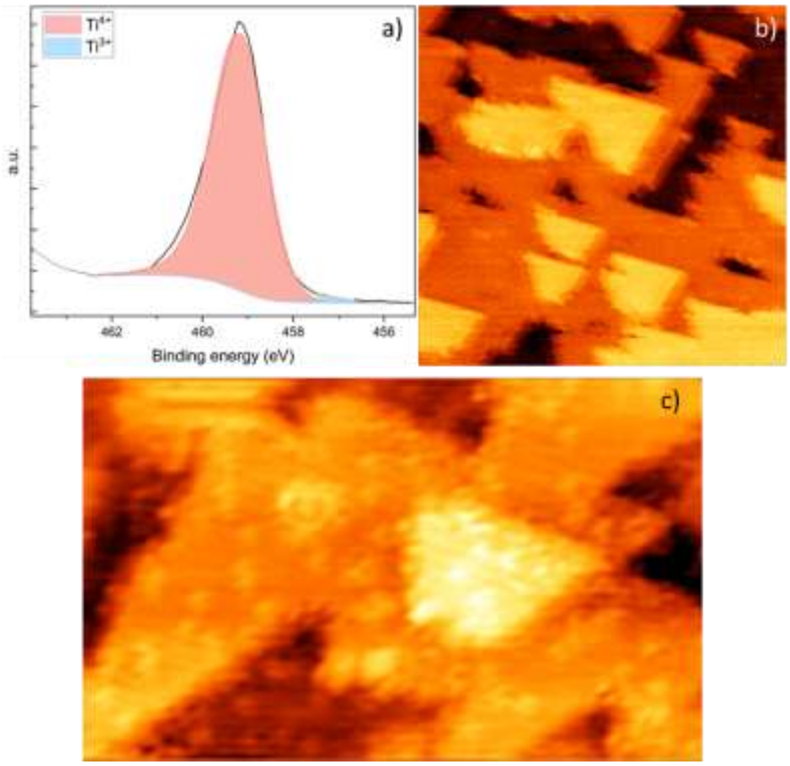

Figure S2. (a) XP spectra of Ti 2p 3/2 region after cysteine evaporation. (b) STM image of clean anatase (101). 35x35 nm; image taken with 1.05 V of bias voltage and 0.3 nA current. (c) STM image of anatase (101) surface measured after dosing 50 L of cysteine at room temperature on a pre-cleaned surface. 20x10 nm; image taken with 1.1 V of bias voltage and 0.1 nA current.

1.7 Oxidation procedure

The sample was transferred out of the XPS chamber through a load-lock connected to the main UHV system. The irradiation procedure was carried out under atmospheric pressure. UV illumination was provided by a commercial lamp with a central wavelength of 365 nm, positioned approximately 10 cm from the sample surface. The irradiance at the sample was estimated to be ~850 $\mu W\cdot cm^{-2}$, corresponding to a photon flux of ~1.5×10^{15} photons$\cdot cm^{-2}\cdot s^{-1}$ at 365 nm. Illumination conditions of this magnitude are comparable to those commonly employed in TiO_2 photocatalytic oxidation studies (~0.5–2 $mW\cdot cm^{-2}$) (*6, 7*).

1.8 Computational details:

Density Functional Theory (DFT) calculations were carried out using the plane-wave-based Quantum ESPRESSO package (*8, 9*). Electron–ion interactions were described with ultrasoft pseudopotentials (*10*), applying energy cutoffs of 52 Ry for the kinetic energy and 575 Ry for the charge density in all simulations. The Perdew–Burke–Ernzerhof (PBE) (*11*) was used for the electron exchange-correlation, and van der Waals interactions were included via the DFT-D3 correction (*12*). A Hubbard U value of 3.9 eV was applied to Ti atoms, following previous literature (*13*–*18*).

The anatase $TiO_2(101)$ surface was modelled using a slab of three O-Ti-O trilayers, and a (1x2) supercell model (defined with respect to the conventional cell) with two Ti_{5c} rows (as shown in **Figure S3a**). The oxygen and titanium atoms in the bottom layer fixed at their bulk positions during geometry optimization to simulate a semi-infinite solid and a vacuum space of at least 25 Å was included in the direction perpendicular to the surface to prevent interactions between adjacent periodic images. All geometry optimizations were performed at the Γ point. Ball-and-stick models were visualized using VESTA software (*19*).

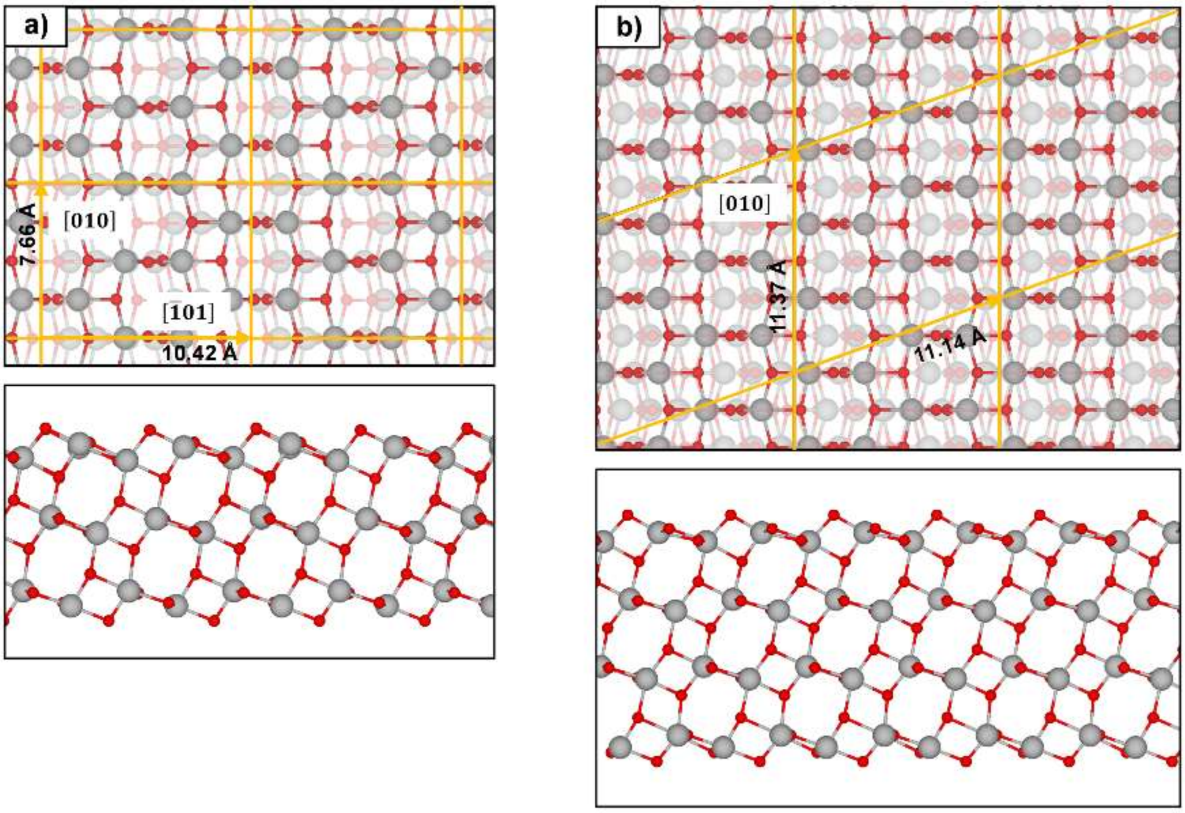

Figure S3. Ball and stick representations (top and side view) of the slab models used to describe the anatase TiO_2(101) surface. Panels (a) and (b) depict the smaller (1 × 2) and larger (2 × 3) slab models, respectively. The two surface supercells are constructed based on the conventional and primitive anatase TiO_2 bulk unit cells, respectively. In the top views, the surface supercell boundaries are highlighted in orange, while the two/three trilayers underneath the surface are shown transparently to enhance visual clarity. The side view is shown along the [010] direction. Color scheme: Ti atoms in grey and O atoms in red

The adsorption energies (ΔE_{ads}) in vacuum for cysteine on the TiO_2(101) surface were calculated using the following formula:

$$\Delta E_{ads} = E_{cys/TiO2} - (E_{cys} + E_{TiO2})$$

where $E_{cys/TiO2}$ is the total energy of the optimized system with an adsorbed cysteine molecule, E_{cys} is the total energy of an isolated cysteine molecule in the gas phase, and E_{TiO2} is the total energy of the optimized TiO_2(101) surface without any adsorbed molecules. For cases where the adsorption of a single cysteine molecule was computed in the presence of water molecules, the adsorption energies were determined as follows:

$$\Delta E_{ads} = E_{cys+nH2O/TiO2} - (E_{cys} + nE_{H2O} + E_{TiO2})$$

where $E_{cys+nH2O/TiO2}$ is the total energy of the system with a cysteine molecule adsorbed on the TiO_2 surface in the presence of n water molecules, and E_{H2O} is the total energy of a water molecule in the gas phase.

XPS core level shifts (CLSs) were calculated within the ΔSCF method (*20*) using pseudopotentials and localized basis sets containing a core 1s hole in the case of C, N, and O and a 2p hole in the case of S. Since this approach does not provide absolute binding energies (BEs), but just their values with respect to a chosen reference, to be able to compare the CLSs of different structures, we added one reference molecule in the simulation cell, notably CH_4, N_2, O_2 or H_2S to calculate the CLS of a C, N, O and S photoexcited atom, respectively. To minimize the interaction of these reference molecules with the TiO2 slab and the adsorbed cysteine, we inserted them in the vacuum region, with a distance of at least 12 Å from any other atom and enlarged the supercell size accordingly. For these simulations, the plane-wave basis set energy cutoffs were increased to 74 and 575 Ry.

The vibrational frequencies of cysteine adsorbed on anatase $TiO_2(101)$ were calculated within the harmonic approximation. Numerical Hessian matrices were constructed using finite atomic displacements (±0.02 a.u.) along the three Cartesian directions from the equilibrium positions, and the corresponding atomic force components were evaluated. The Hessian matrices were subsequently diagonalized at the Γ point to obtain the vibrational frequencies associated with the normal modes. To decouple the motion of relaxed atoms from that of atoms constrained to their bulk positions, the Hessian matrix elements involving constrained atoms were set to zero.

The photooxidation mechanism of cysteine was investigated using DFT calculations performed with the CRYSTAL17 package, in which Kohn–Sham orbitals are expanded in localized Gaussian-type basis functions. All-electron basis sets were employed throughout: Ti 86-411(d41) and O 8-411(d1) for the TiO_2 slab, and H 511(p1), C 6-311(d11), and O 8-411(d11) for cysteine and molecular O_2. To accurately describe the reaction intermediates, all calculations were carried out using the range-separated hybrid Heyd–Scuseria–Ernzerhof functional (HSE06). Long-range dispersion interactions were accounted for by including Grimme's D3 corrections (HSE06+D3), allowing the evaluation of van der Waals effects relative to the pure HSE06 functional. The cutoff thresholds for the evaluation of Coulomb and exchange series in the self-consistent field (SCF) procedure were set to 10^{-7} for Coulomb overlap, Coulomb penetration, exchange overlap, and exchange pseudo-overlap in direct space, and to 10^{-14} for exchange pseudo-overlap in reciprocal space. SCF convergence was achieved when the total energy difference between consecutive cycles was below 10^{-6} hartree.

For these calculations, the anatase $TiO_2(101)$ surface was modelled using a slab of four O–Ti–O trilayers and a (2×3) supercell (defined with respect to the primitive cell) containing two Ti_{5c} rows (as shown in **Figure S3b**). The oxygen and titanium atoms in the bottom layer were fixed at their bulk positions during geometry optimization to simulate a semi-infinite solid. Brillouin-zone sampling was performed using a 2×2 Monkhorst–Pack k-point grid, and spin polarization was included when required. Geometry optimizations were considered converged when both the root-mean-square and maximum values of the energy gradients and atomic displacements satisfied the default CRYSTAL17 convergence thresholds. Specifically, maximum and rms forces were set to 4.50×10^{-4} and 3.00×10^{-4} au, respectively, while maximum and rms atomic displacements were set to 1.80×10^{-3} and 1.20×10^{-3} au, respectively.

The photoexcitation was modelled by forcing the system (TiO_2 with adsorbed cysteine) in the triplet state, effectively simulating a photoexcited electron-hole pair. In all calculations, the cysteine and O_2 molecules were adsorbed exclusively on the upper surface of the TiO_2 slab, while titanium and oxygen atoms in the bottom layer were kept fixed at their bulk positions during geometry optimization.

S.I. 2 Optimized adsorption configuration on the anatase TiO_2(101) surface

2.1 Molecular Geometries:

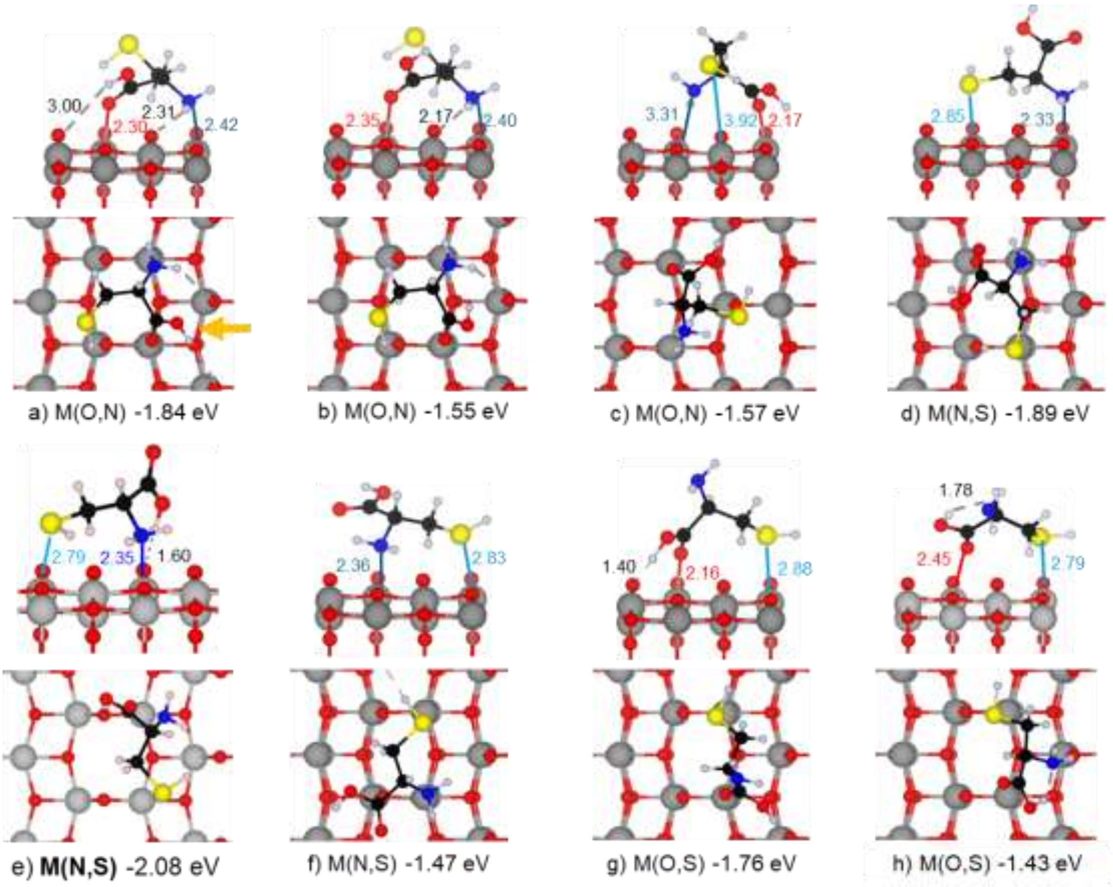

Figure S4. Side and top views of the optimized structures of molecular cysteine adsorption configurations on the anatase TiO_2(101) surface, calculated using QE with the PBE+D3+U approach. Gray, red, blue, yellow, white, and black spheres represent Ti, O, N, S, H, and C atoms, respectively. Dashed lines indicate hydrogen bonds. Adsorption energies (in eV) are reported below each structure, and selected bond lengths (in Å) are indicated. For clarity, only a portion of the supercell is shown.

2.2 Deprotonated Geometries:

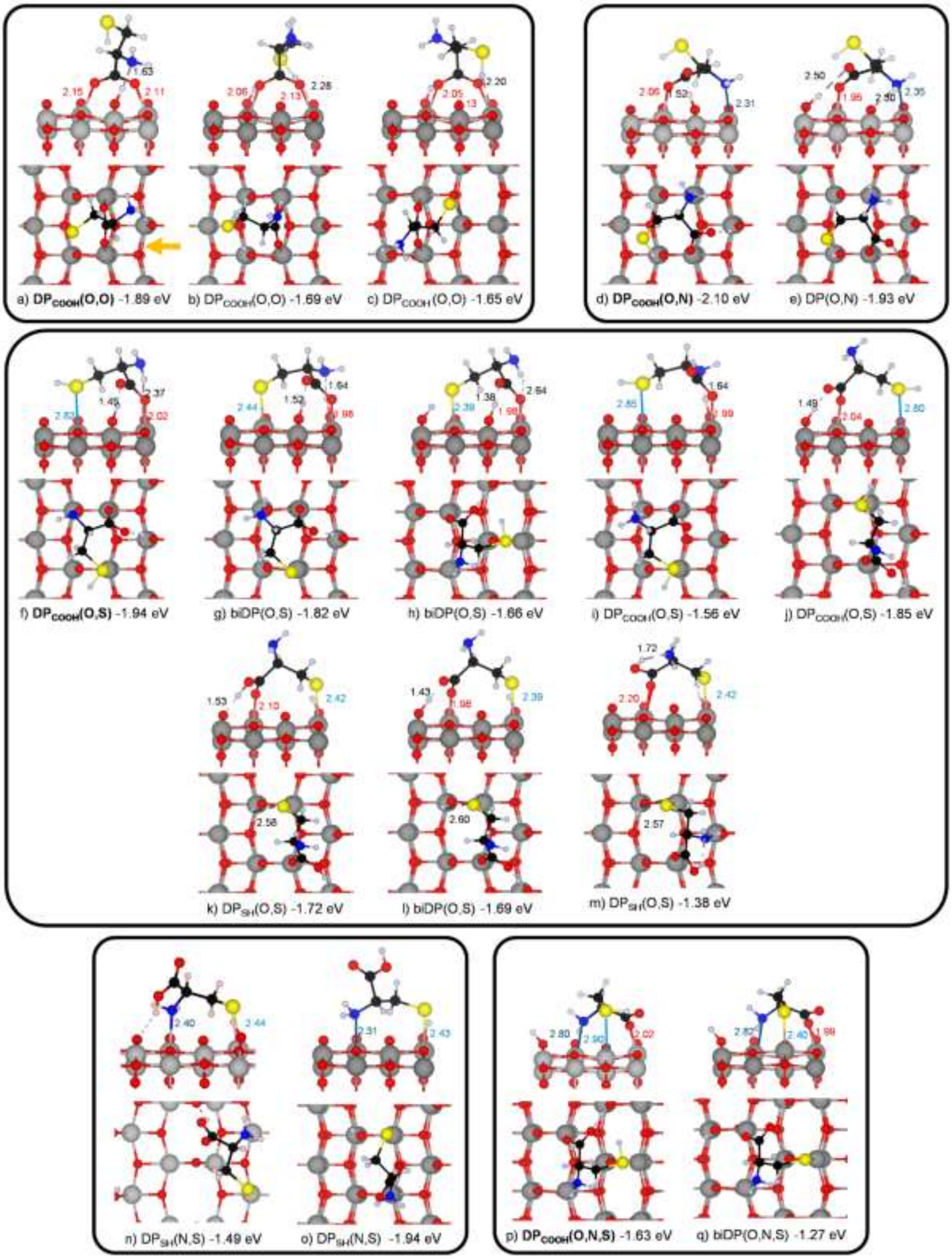

Figure S5. Side and top views of the optimized structures of deprotonated cysteine adsorption configurations on the anatase TiO_2(101) surface, calculated using QE with the PBE+D3+U approach. Gray, red, blue, yellow, white, and black spheres represent Ti, O, N, S, H, and C atoms, respectively. Dashed lines indicate hydrogen bonds. Adsorption energies (in eV) are reported below each structure, and selected bond lengths (in Å) are indicated. For clarity, only a portion of the supercell is shown.

2.3 Zwitterionic Geometries:

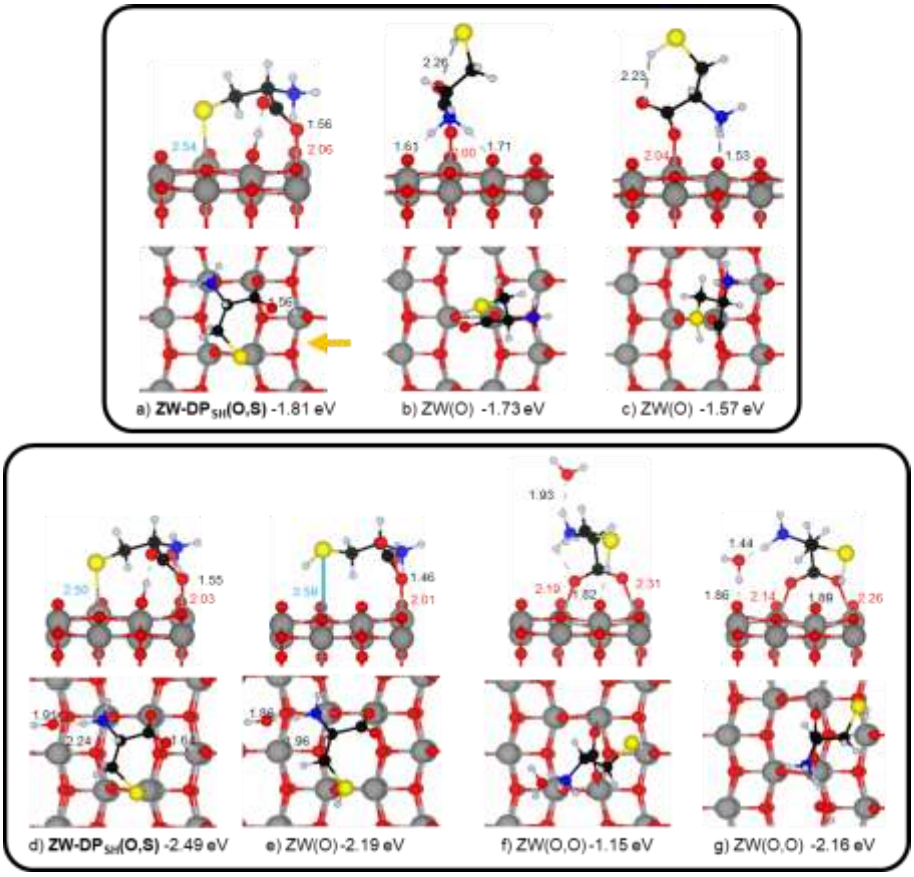

Figure S6. Side and top views of the optimized structures of zwitterionic cysteine adsorption configurations on the anatase TiO_2(101) surface, calculated using QE with the PBE+D3+U approach. The top and bottom panels show the structures without and with one gas-phase water molecule, respectively. Gray, red, blue, yellow, white, and black spheres represent Ti, O, N, S, H, and C atoms, respectively. Dashed lines indicate hydrogen bonds. Adsorption energies (in eV) are reported below each structure, and selected bond lengths (in Å) are indicated. For clarity, only a portion of the supercell is shown.

2.4 Energy comparison between Geometries:

Table S1. Comparison of the relative energetic stability of the most stable molecular, deprotonated, and zwitterionic cysteine adsorption configurations. Calculations were performed using QE with PBE+U+D3 and CRY17 with HSE06+D3 (values in parentheses). Values in square brackets are taken from Pantaleone et al. for comparison with the present results (*21*). A hyphen indicates that the corresponding configuration was not found.

**QE/PBE+U+D3 (CRY17/HSE06+D3)**	**Figure**	**ΔE (eV)**	**ΔE_{ads} (eV)**
DP(O,N)	Figure 4c	0.00 (0.00) [+0.12]	-2.10 (-2.06)
M(N,S)	Figure 4a	+0.02 (+0.07) [+0.17]	-2.08 (-1.99)
ZW-DP$_{SH}$(O,S)	Figure 5a	+0.29 (+0.39)	-1.81 (-1.66)
ZW(O,O)		− (+0.29) [0.00]	− (-1.77)

S.I. 3 Core Level shifts and XP fitting information

3.1 Core level shifts information:

Table S2. CLSs (in eV) calculated for selected adsorption configurations and the isolated molecule for different photoexcited core electrons, each with respect to its reference.

		Scheme 1	Fig. 4a	Fig. 4b	Fig. 4c	Fig. 4d	Fig. 4e	Fig. 5a
**Edge**	**Atom type**	**Cys gas-phase**	**M(N,S)**	**DP_{COOH}(O,O)**	**DP_{COOH}(O,N)**	**DP_{COOH}(O,S)**	**DP_{COOH}(O,N,S)**	**ZW-DP_{SH}(O,S)**
**C 1s**	**C–O**	+3.7	+3.1	+3.0	+3.0	+2.8	+2.9	+3.0
	**C–N**	+1.3	+1.5	+1.1	+1.1	+0.9	+1.0	+1.9
	**C–S**	+0.8	+0.8	+0.7	+0.2	+0.2	+0.5	+0.4
**N 1s**	**NH_2**	-2.3	–	-0.8	–	-1.8	–	–
	**NH_2–Ti_{5c}**	–	-0.7	–	-0.7	–	-1.5	–
	**NH_3^+**	–	–	–	–	–	–	+1.7
**O 1s**	**O1–Ti**	–	–	-0.6	-0.9	-1.0	-1.1	-0.7
	**O2–Ti**	–	–	-0.6	-1.1	-1.0	-1.2	-0.6
	**C–OH/ C=O**	+1.1/-1.0	-0.1/-1.4	–	–	–	–	–
**S 2p**	**S–H**	-0.5	-0.4	-0.5	-0.9	-0.6	-0.8	–
	**S–Ti**	–	–	–	–	–	–	-2.5

3.2 Peak fitting protocol:

For each core level, deconvolution started with the minimum set of chemically plausible components; an additional peak was introduced only when demanded by either the envelope shape or the residual trace.

C 1s: L-cysteine has three inequivalent carbon atoms (C-SH, C-NH_2, COOH), so three components were fitted. A fourth peak (C-SO_x) was added only when necessary. During UV exposure at room temperature the C-S peak shifts towards lower binding energies while growing, indicating the deposition of adventitious carbon

O 1s: On clean TiO_2(101), the line consists of a lattice oxygen peak plus a shoulder from intrinsic defects. Adsorption leaves extra intensity at high binding energy. Adding one component assigns this to carboxylate oxygens and removes the mismatch.

S 2p: The S 2p level is a doublet ($2p_{3/2}$–$2p_{1/2}$, 1.2 eV split, 2 : 1 area). A single -SH doublet left a low BE shoulder. Introducing a second doublet assigned to surface bound thiolate (S-Ti) eliminates the shoulder and flattens the residual. After illumination a new high binding energy doublet appears at 168.7 eV, which is assigned to fully oxidized sulfur species (sulfonate like, –SO_3H). However, this component alone does not adequately reproduce the experimental line shape. An additional intermediate doublet at 166.5 eV is required.

N 1s: A one peak fit produced an overly broad, asymmetric envelope. Splitting into neutral $-NH_2$ and protonated $-NH_3^+$ components removed the asymmetry and gave a satisfactory residual.

Table S3. Results of the fitting for XPS data All peaks have been fitted using a Shirley background and a Asymmetric Lorentzian lineshape LA(30) that has been proofed adequate for lightly conductive oxides (*22–24*).

Edge	Surface	Atom type	Position (eV)	FWHM (eV)	Ratio	Residual STD
O 1s	Clean	Intrinsic	531.0	1.5	0.3	2.3
		Lattice	530.3	1.2	1	
	Adsorption	Intrinsic	531.1	1.2	0.3	1.9
		Carboxylate	532.0	1.4	0.1	
	Oxidation (30 min)	Intrinsic	531.0	1.3	0.35	1.5
		Carboxylate	531.9	1.6	0.1	
Ti 2p	Clean	Ti4+	549.1	1.1	1	3.1
		Ti3+	457.3	1.5	0.05	
	Adsorption	Ti4+	459.1	1.3	1	3.1
		Ti3+	457.3	1.0	0.02	
	Oxidation (30 min)	Ti4+	459.1	1.2	1	3.2
		Ti3+	457.3	1.2	0.02	
C 1s	Adsorption	C-O	289.0	2.1	1	1.0
		C-N	285.9	1.7	1.31	
		C-S	285.2	1.8	1.75	
	Oxidation (5 min)	C-O	288.9	1.8	1	0.2
		C-N	285.8	1.5	1.5	
		C-S/C-Hx	284.8	1.4	2.5	
		C-SOx	286.6	1.8	0.6	
	Oxidation (30 min)	C-O	288.8	2.1	1.0	0.3
		C-N	285.2	1.6	1.7	
		C-S/C-Hx	284.6	1.5	2.5	
		C-SOx	286.5	1.7	1.0	
S $2p_{3/2}$	Adsorption	S-H	163.9	1.5	1	1.0
		S-Ti	161.6	1.1	0.4	
	Oxidation (5 min)	S-H	163.9	1.4	1	1.0
		S-Ti	161.7	1.7	0.6	
		S-Ox	166.5	1.1	0.6	
		SO_3H	168.7	0.9	0.4	
	Oxidation (30 min)	SO_3H	168.7	1.8		0.8
N 1s	Adsorption	NH2	400.3	1.5	1	0.5
		NH3+	402.2	1.5	0.6	
	Oxidation (30 min)	NH2	400.2	1.8	1	0.7
		NH3+	402.0	2.0	0.53	

S.I. 4 XPS Dark experiment

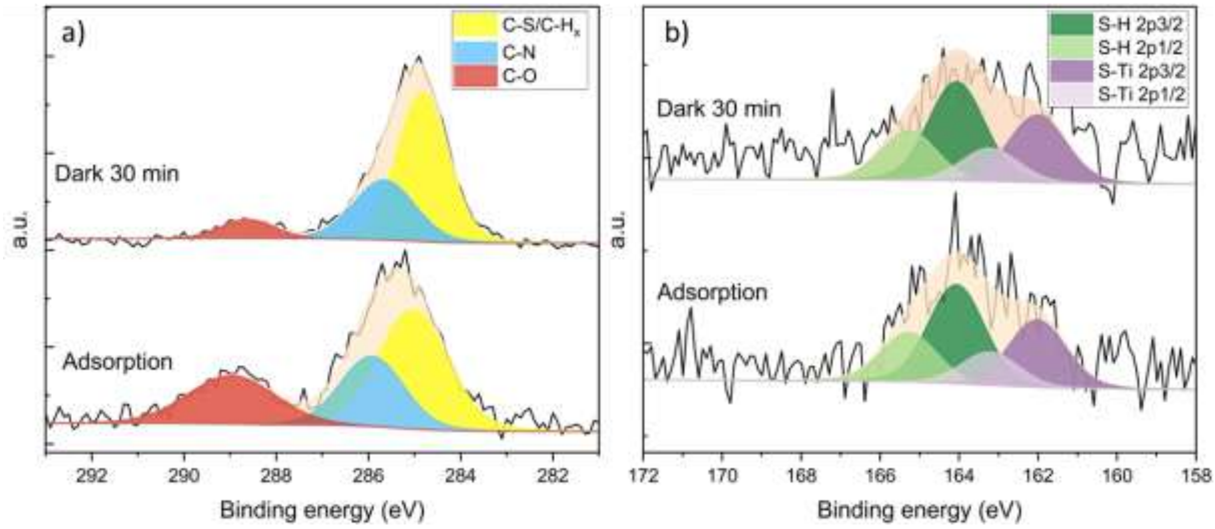

Figure S7. Deconvoluted XP spectra of adsorbed cysteine on anatase TiO_2(101) under 30 min of atmospheric exposure in dark conditions. Experimental data (black lines) and deconvoluted components (coloured areas) are presented for (a) C 1s, (b) S 2p, before and after exposure at room temperature to atmospheric pressure.

S.I. 5 XPS O 1s after UV

The O 1s spectra recorded before irradiation and after 30 min of UV exposure are shown in Figure S7. No significant spectral changes are observed upon irradiation. The O 1s signal is dominated by lattice oxygen from TiO_2, which masks the comparatively small contribution from oxygen containing oxidation products. Therefore, the O 1s region does not provide additional insight into the progression of cysteine photooxidation.

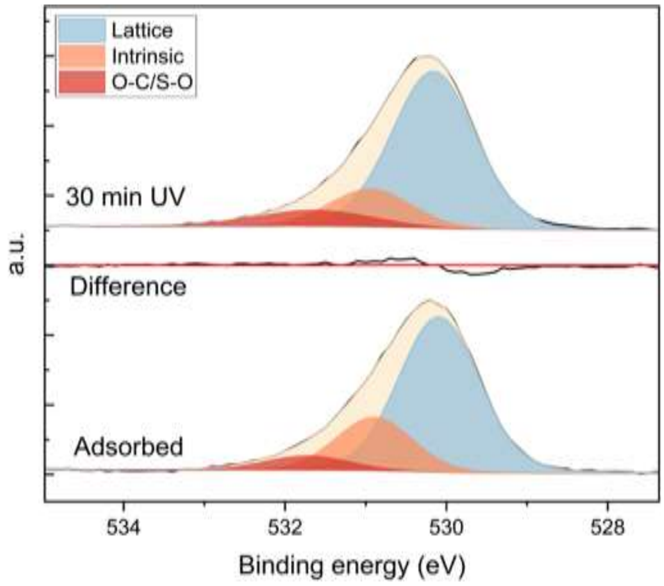

Figure S8. Deconvoluted XP spectra of adsorbed cysteine on anatase TiO_2(101) under UV irradiation. Experimental data (black lines) and deconvoluted components (colored areas) are presented for O 1s spectra. Difference spectra (30 min UV - adsorbed) illustrate the absence of relevant spectral changes.

S.I. 6 Calculation of reaction intermediates

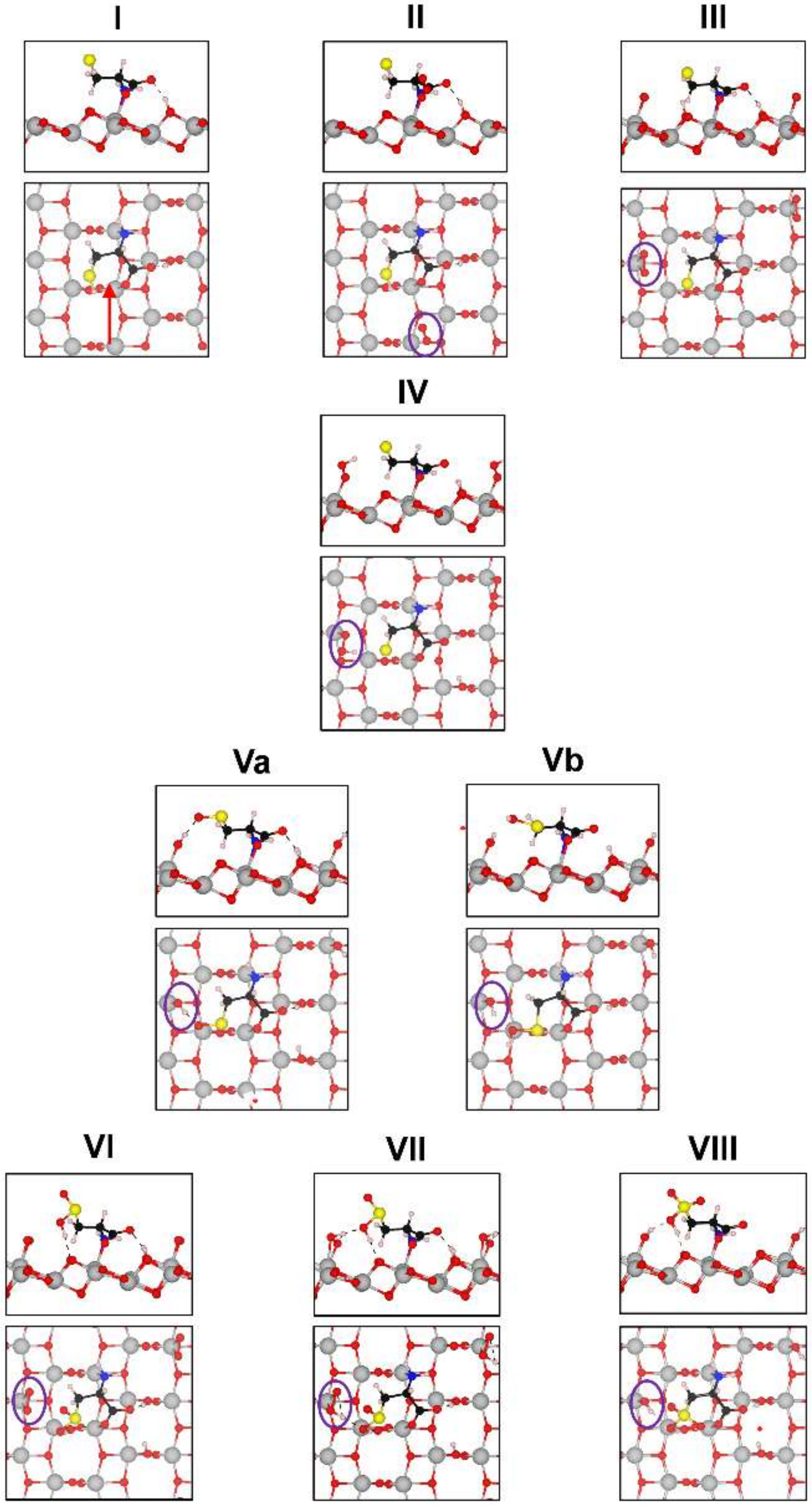

Figure S9. Ball-and-stick representation (top and side view) of the reaction intermediates shown in Figure 7 along the photooxidation pathway. Grey, red, blue, yellow, white, and black spheres represent Ti, O, N, S, H, and C atoms, respectively. Dashed lines indicate hydrogen bonds. For clarity, only a portion of the surface supercell is shown. Adsorbed oxygen moieties are highlighted with a purple circle. The red arrow indicates the viewing direction of the side view.

S.I. 7 Calculation of amino group photooxidation

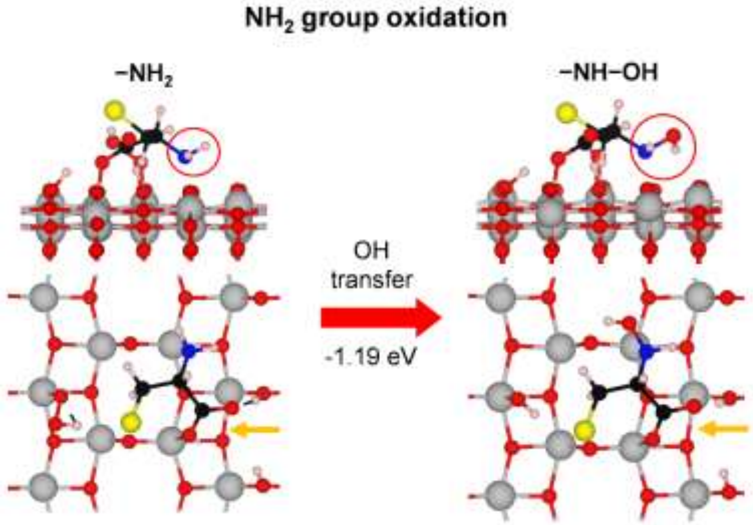

Figure S10. Side and top views of the optimized structures illustrating the reaction pathway in which the –NH_2 group (left) is oxidized to –NH–OH (right) after formation of the superoxo/hydroperoxo intermediate. The structures are calculated using Quantum ESPRESSO with the PBE+D3+U approach. Gray, red, blue, yellow, white, and black spheres represent Ti, O, N, S, H, and C atoms, respectively. For clarity, only a portion of the supercell is shown. The –NH_2 group undergoing oxidation is highlighted with a red circle.

S.I. 8 Calculation of S 2p Shifts Along the Photooxidation Pathway.

To further corroborate the intermediates proposed by the HSE06+D3 reaction pathway calculations, we estimated the relative energetic position of sulphur 2p levels for each intermediate, spanning formal sulphur oxidation states from -2 (thiol) to +4 (sulfonic acid). For these calculations, a localized all electron basis set as implemented in the CRYSTAL17 code was employed. Under these conditions, the pseudo core-hole approach used for the adsorption CLS calculations is not applicable.

Therefore, sulphur 2p level trends were extracted from the projected density of states (PDOS) onto S-derived orbitals for each intermediate. Because all slab calculations were performed with identical settings and referenced to a common vacuum level within the 2D-periodic CRYSTAL17 framework, direct comparison of the relative S derived level positions across intermediates is meaningful. The resulting values and the derived relative shifts are summarized in Table 2 in the main text.

Increased sulphur oxidation stabilizes the S derived electronic levels (shifting them to lower energies in the PDOS representation); when expressed as core-level binding energies relative to a common reference, this corresponds to the experimentally observed shift of the S 2p peak toward higher binding energy with increasing oxidation state.

References

1. Setvín, M.; Daniel, B.; Mansfeldova, V.; Kavan, L.; Scheiber, P.; Fidler, M.; Schmid, M.; Diebold, U. Surface preparation of TiO2 anatase (101): Pitfalls and how to avoid them. *Surface Science* **2014,** *626*, 61–67. DOI: 10.1016/j.susc.2014.04.001.
2. Stierle, A.; Keller, T. F.; Noei, H.; Vonk, V.; Roehlsberger, R. DESY NanoLab. *JLSRF* **2016,** *2*, A76. DOI: 10.17815/jlsrf-2-140.

3. Powell, C. J.; Jablonski, A. The NIST Electron Effective-Attenuation-Length Database. *JSA* **2002,** *9* (3), 322–325. DOI: 10.1384/jsa.9.322.

4. Jürgensen, A.; Raschke, H.; Esser, N.; Hergenröder, R. An in situ XPS study of L-cysteine co-adsorbed with water on polycrystalline copper and gold. *Applied Surface Science* **2018,** *435*, 870–879. DOI: 10.1016/j.apsusc.2017.11.150.

5. Ching, C. B.; Hidajat, K.; Uddin, M. S. Evaluation of Equilibrium and Kinetic Parameters of Smaller Molecular Size Amino Acids on KX Zeolite Crystals via Liquid Chromatographic Techniques: Amino acid sizes. *Separation Science and Technology* **1989,** *24* (7-8), 581–597. DOI: 10.1080/01496398908049793.

6. Berger, T. E.; Regmi, C.; Schäfer, A. I.; Richards, B. S. Photocatalytic degradation of organic dye via atomic layer deposited TiO2 on ceramic membranes in single-pass flow-through operation. *Journal of Membrane Science* **2020,** *604*, 118015. DOI: 10.1016/j.memsci.2020.118015.

7. Kohantorabi, M.; Ugolotti, A.; Sochor, B.; Roessler, J.; Wagstaffe, M.; Meinhardt, A.; Beck, E. E.; Dolling, D. S.; Garcia, M. B.; Creutzburg, M.; Keller, T. F.; Schwartzkopf, M.; Vayalil, S. K.; Thuenauer, R.; Guédez, G.; Löw, C.; Ebert, G.; Protzer, U.; Hammerschmidt, W.; Zeidler, R.; Roth, S. V.; Di Valentin, C.; Stierle, A.; Noei, H. Light-Induced Transformation of Virus-Like Particles on TiO2. *ACS applied materials & interfaces* **2024,** *16* (28), 37275–37287. DOI: 10.1021/acsami.4c07151.

8. Giannozzi, P.; Baroni, S.; Bonini, N.; Calandra, M.; Car, R.; Cavazzoni, C.; Ceresoli, D.; Chiarotti, G. L.; Cococcioni, M.; Dabo, I.; Dal Corso, A.; Gironcoli, S. de; Fabris, S.; Fratesi, G.; Gebauer, R.; Gerstmann, U.; Gougoussis, C.; Kokalj, A.; Lazzeri, M.; Martin-Samos, L.; Marzari, N.; Mauri, F.; Mazzarello, R.; Paolini, S.; Pasquarello, A.; Paulatto, L.; Sbraccia, C.; Scandolo, S.; Sclauzero, G.; Seitsonen, A. P.; Smogunov, A.; Umari, P.; Wentzcovitch, R. M. QUANTUM ESPRESSO: a modular and open-source software project for quantum simulations of materials. *J. Phys.: Condens. Matter* **2009,** *21* (39), 395502. DOI: 10.1088/0953-8984/21/39/395502}.

9. Giannozzi, P.; Andreussi, O.; Brumme, T.; Bunau, O.; Buongiorno Nardelli, M.; Calandra, M.; Car, R.; Cavazzoni, C.; Ceresoli, D.; Cococcioni, M.; Colonna, N.; Carnimeo, I.; Dal Corso, A.; Gironcoli, S. de; Delugas, P.; DiStasio, R. A.; Ferretti, A.; Floris, A.; Fratesi, G.; Fugallo, G.; Gebauer, R.; Gerstmann, U.; Giustino, F.; Gorni, T.; Jia, J.; Kawamura, M.; Ko, H.-Y.; Kokalj, A.; Küçükbenli, E.; Lazzeri, M.; Marsili, M.; Marzari, N.; Mauri, F.; Nguyen, N. L.; Nguyen, H.-V.; Otero-de-la-Roza, A.; Paulatto, L.; Poncé, S.; Rocca, D.; Sabatini, R.; Santra, B.; Schlipf, M.; Seitsonen, A. P.; Smogunov, A.; Timrov, I.; Thonhauser, T.; Umari, P.; Vast, N.; Wu, X.; Baroni, S. Advanced capabilities for materials modelling with Quantum ESPRESSO. *J. Phys.: Condens. Matter* **2017,** *29* (46), 465901. DOI: 10.1088/1361-648X/aa8f79}.

10. Dal Corso, A. Pseudopotentials periodic table: From H to Pu. *Computational Materials Science* **2014,** *95*, 337–350. DOI: 10.1016/j.commatsci.2014.07.043.

11. Perdew, J. P.; Burke, K.; Ernzerhof, M. Generalized Gradient Approximation Made Simple. *Physical review letters* **1996,** *77* (18), 3865–3868. DOI: 10.1103/PhysRevLett.77.3865}.

12. Grimme, S.; Antony, J.; Ehrlich, S.; Krieg, H. A consistent and accurate ab initio parametrization of density functional dispersion correction (DFT-D) for the 94 elements H-Pu. *The Journal of Chemical Physics* **2010,** *132* (15), 154104. DOI: 10.1063/1.3382344.

13. Hu, Z.; Metiu, H. Choice of U for DFT+ U Calculations for Titanium Oxides. *J. Phys. Chem. C* **2011,** *115* (13), 5841–5845. DOI: 10.1021/jp111350u.

14. Selcuk, S.; Zhao, X.; Selloni, A. Structural evolution of titanium dioxide during reduction in high-pressure hydrogen. *Nature materials* **2018,** *17* (10), 923–928. DOI: 10.1038/s41563-018-0135-0.
15. Wang, Y.; Wen, B.; Dahal, A.; Kimmel, G. A.; Rousseau, R.; Selloni, A.; Petrik, N. G.; Dohnálek, Z. Binding of Formic Acid on Anatase TiO 2 (101). *J. Phys. Chem. C* **2020,** *124* (37), 20228–20239. DOI: 10.1021/acs.jpcc.0c06031.
16. Setvin, M.; Hulva, J.; Wang, H.; Simschitz, T.; Schmid, M.; Parkinson, G. S.; Di Valentin, C.; Selloni, A.; Diebold, U. Formaldehyde Adsorption on the Anatase TiO2 (101) Surface: Experimental and Theoretical Investigation. *J. Phys. Chem. C* **2017,** *121* (16), 8914–8922. DOI: 10.1021/acs.jpcc.7b01434.
17. Setvin, M.; Shi, X.; Hulva, J.; Simschitz, T.; Parkinson, G. S.; Schmid, M.; Di Valentin, C.; Selloni, A.; Diebold, U. Methanol on Anatase TiO2 (101): Mechanistic Insights into Photocatalysis. *ACS Catal.* **2017,** *7* (10), 7081–7091. DOI: 10.1021/acscatal.7b02003.
18. Selcuk, S.; Selloni, A. Facet-dependent trapping and dynamics of excess electrons at anatase TiO2 surfaces and aqueous interfaces. *Nature materials* **2016,** *15* (10), 1107–1112. DOI: 10.1038/nmat4672.
19. Momma, K.; Izumi, F. VESTA 3 for three-dimensional visualization of crystal, volumetric and morphology data. *J Appl Crystallogr* **2011,** *44* (6), 1272–1276. DOI: 10.1107/S0021889811038970.
20. García-Gil, S.; García, A.; Ordejón, P. Calculation of core level shifts within DFT using pseudopotentials and localized basis sets. *Eur. Phys. J. B* **2012,** *85* (7). DOI: 10.1140/epjb/e2012-30334-5.
21. Pantaleone, S.; Rimola, A.; Sodupe, M. Canonical, Deprotonated, or Zwitterionic? A Computational Study on Amino Acid Interaction with the TiO2 (101) Anatase Surface. *J. Phys. Chem. C* **2017,** *121* (26), 14156–14165. DOI: 10.1021/acs.jpcc.7b03305.
22. Engelhard, M. H.; Baer, D. R.; Herrera-Gomez, A.; Sherwood, P. M. A. Introductory guide to backgrounds in XPS spectra and their impact on determining peak intensities. *Journal of Vacuum Science & Technology A: Vacuum, Surfaces, and Films* **2020,** *38* (6). DOI: 10.1116/6.0000359.
23. Major, G. H.; Fairley, N.; Sherwood, P. M. A.; Linford, M. R.; Terry, J.; Fernandez, V.; Artyushkova, K. Practical guide for curve fitting in x-ray photoelectron spectroscopy. *Journal of Vacuum Science & Technology A* **2020,** *38* (6). DOI: 10.1116/6.0000377.
24. Shard, A. G. Practical guides for x-ray photoelectron spectroscopy: Quantitative XPS. *Journal of Vacuum Science & Technology A* **2020,** *38* (4). DOI: 10.1116/1.5141395.
